# Supplementary material for: Comparative optical genome analysis of two pangolin species: Manis pentadactyla and Manis javanica
Source: Gigascience. 2016 Dec 29;5(1):1–5. doi: 10.1093/gigascience/giw001 (PMC6082621; doi:10.1093/gigascience/giw001)
Supplement: Supplementary file 1 [file giw001_giga-d-16-00053_original-submission.pdf]

Manuscript
Click here to download Manuscript Main manuscript text.docx
Click here to view linked References

Data Note

Title: Comparative optical genome analysis of two Pangolin species *Manis pentadactyla* and *Manis javanica*

Authors: Huang Zhihai[1,*,&], Xu Jiang[2,&], Xiao Shuiming[2,&], Liao Baosheng[2,3,&], Gao Yuan[4], Zhai Chaochao[3], Qiu Xiaohui[1], Xu Wen[1], Chen Shilin[2,*]

1 Guangdong Provincial Hospital of Chinese Medicine, Guangzhou 510006, China

2 Institute of Chinese Materia Medica, China Academy of Chinese Medicines, Beijing 100700, China

3 Ultravision-tech, Beijing 100089, China

4 Institute of Medicinal Plant Development, Chinese Academy of Medical Sciences, Beijing 100193, China

* Correspondence: Huang Zhihai, zhhuang7308@163.com; Chen Shilin, slchen@icmm.ac.cn.

& Contributed to this paper equally.

Abstract:

Background: Pangolin is a Pholidota mammal with large keratin scales protecting its skin, two species of which-*Manis pentadactyla* and *Manis javanica*-have been recorded as critically endangered on the IUCN Red List of Threaten Species. Optical mapping constructs high-solution restriction maps from single stained DNA molecule, then it allows the analysis of genome at mega-base scale as well as the assistance of genome assembly. Here, we constructed the restriction maps of *M. pentadactyla* and *M. javanica* using optical mapping hoping to help these species genome assembly and analysis.

Findings: Genomic DNA was nicked with Nt.BspQI followed by labeling use certain fluorescent labeled bases, which detected in the Irys optical mapping system. Totally, 3,313,734 DNA molecules (517.847 Gb) for *M. pentadactyla* and 3,439,885 DNA molecules (504.743 Gb) for *M.javanica* were obtained, which about 178X and 177X genome coverage respectively. Qualified molecules (≥150 Kb and the label density > 6 sites/100 kilobases) were analyzed using *de novo* assembly program embedded in IrysView pipeline. Two maps with genome size 2.91 Gb and 2.85 Gb were obtained, the maps N50 are 1.88 Mb and 1.97 Mb respectively.

Conclusions: Optical mapping revealed large-scale structural information especially for the non-model genome that without a good reference. Further, it holds the potential for the guidance of NGS-based *de novo* assembly. Our data provides a resource for Manidae genome analysis and references for *de novo* assembly. This note also implys a new sight for Manidae evolutionary analysis at the genome-structure level.

Key words: optical mapping, restriction maps, pangolin, Manidae

Data description:

Pangolin, sole representatives the order Pholidota, is a group of nocturnal mammals well-known by their full armor of scales. Around the world, pangolin only contains eight species, and these species have been distributed to three genera-Manis, Phataginus and Smutsia according to Gaudin *et al* [1]. *Manis pentadactyla* and *Manis javanica* belong to the Asian subfamily of Pangolin, these of which have long been used as an ingredient traditional medicine in China and South-east Asia. Due to poaching and deforestation, their colony and habit have been largely destroyed and these two species are on the verge of extinction. Now *M. pentadactyla* and *M. javanica* have been recorded as critically endangered on the IUCN Red List of Threaten Species.

Optical mapping is a molecular tool for chromosome-wide restriction maps production [2]. During the optical mapping process, stretched linear DNA were labeled at specific sequence motives then were exposed under fluorescence microscope for generating image signal, which translated to motif-distance information for further analysis [3, 4]. Unlike traditional sequencing approaches, optical mapping possesses several advantages such as single molecule analysis and long DNA molecules, which can be used for *de novo* maps assembly or sequencing contigs anchoring. So far, optical mapping have facilitated or improved a large arrays of genome assemblies including Amborella [5], goat [6], *Oropetium thomaeum* [7] and *Ganoderm lucidum* [8]. Beyond the guide of genome assembly, optical mapping offered a complementary sight for sequence variation analysis in large-region comparison instead of nucleotide matches, which provides several unique traits for evolutionary or functional analysis. Direct comparison between optical maps are usually

conducted in microbes, but it has been lacking so far in large genome like animals or plants [9]. Here we presentative two optical maps of Manis, in order to reveal their genetic structure, we also compare them by pairwise sequence alignments to seek their interspecific variations.

High molecular weight DNA was isolated from *M. pentadactyla* and *M. javanica* blood sample. To be specific, 3 ml blood was orbital sampled and anticoagulated by EDTA, then shipped on ice. For each 3 ml sample, 9 ml RBC lysis solution were mixed and rocked gently at room temperature for 10 min. The mixture were spun at 2000 g at 4 °C for 2 min, then the supernatant were discarded. The pellet was suspended in 3 ml PBS buffer. After remove the insoluble particulates, the mixture were spun again. The supernatant were discarded and the pellet were resuspened in 563 μl refrigerated cell suspension buffer, the cell number in the mixture should be ~0.5×10^7 cells/ml. For gel casting, per 75 μl resuspened buffer was mixed with 25 μl preheated 2% agarose, the gels were solidified at 4 °C for 45 min. Gels casts were immersed in 5 ml of lysis buffer (0.5 M EDTA, pH 9.5; 1 % lauroyl sarcosine, sodium salt; proteinase K, 2 mg/ml) at 50 °C for 2 days for cell lysis. The cell-lysed gels were washed with 1X TE, and immobilized DNA were recovered by melting the gels at 70 °C for 10 min, followed by incubation with GELase (Epicenter) at 43 °C for 45 min. The recovered DNA were drop dialyzed against TE for 4 h using 0.1 μm membrane. Dialyzed DNA were quantified and stored for nicking.

Before molecular nicking, DNA was equilibrated at room temperature for 30 min and gently mixed with wide bore tips. In a 10 μl reaction system, 300 ng equilibrated DNA with 7 unit Nt.BspQI (NEB) nickase and 1 μl nicking buffer were added and mixed. The nicking process was conducted in a thermal cycler at 37 °C for 2 h. The nicked DNA was added with

5 μl labeling master mix which contained 1.5 μl labeling buffer (Bionano), 1.5 μl labeling mix (Bionano), 1 μl Taq polymerase (NEB) for flagging certain motives. The labeling process was conducted at 72 °C for 1 h. Each labeled DNA solution was mixed with 15 μl Repair Master Mix which contained 0.5 μl 10 Thermo polymerase buffer (NEB), 0.4 μl 50X repair mix (Bionano), 0.4 μl 50 mM NAD+ (NEB), 1.0 μl Taq DNA polymerase (NEB), 2.7 μl ultrapure water for nicks repair. The repairing reaction was conduct at 37 °C for 30 mins following by add 1 μl stop solution (Bionano) to stop the reaction. After ligated the nicks, the backbone of the labelled DNA was stained with IrysPrep DNA stain solution (Bionano) at 4 °C overnight. Prepared samples were loaded on Irys chips (Bionano) then full filled the nano-channels in chip, the concentration time was set to 400 s in order to avoid over-staining or over-loading. Fluorescently labelled DNA were illuminated by certain laser, and the signal was caught by an onboard EM CCD camera (Fig. 1). The acquired images were converted to digital data by Auto-detect software. Totally, 517.874 Gb and 504.743 Gb date were generated for *M. pentadactyla* and *M. javanica*, represented 178X and 177X of their predicted genome respectively.

All the data were filtered by IrysView under following criteria: molecule length ≥150 Kb, label signal/noise ratio (SNR) ≥ 3. The number of filtered molecules is about 1,360,730 for *M. pentadactyla* with N50 length of 275.5 Kb and 1,254,380 for *M. javanica* with N50 length of 281.1 Kb. The label density is 10.193/100 Kb for *M. pentadactyla* and 10.151/100 Kb for *M. javanica*. The distance between adjacent labels ranged from 0 Kb to 833.609 Kb for *M. pentadactyla* and 0 Kb to 955.352 Kb for *M. javanica*. In the detecting process, two label sites which are near to each other will be detected as one or can't not be separated, so the distance

of these sites will be set to 0 bp. Simple tandem repeat area, whose repeat unit only has one restriction site, were detected by molecules. The statistics revealed that most of the appeared simple tandem repeat unit size is 4.3 Kb, and the second abundant repeat size is 5.2 Kb in *M. pentadactyla*, and 4.6 Kb and 3.4 Kb in *M. javanica*. The total length of the repeat region accounted for 0.55% and 0.45% of raw data. RefAligner and Assembler packages in IrysView were invoked for *de novo* assembly. The assemble process comprised by molecules pairwise comparison, graph building and maps refinement. In this work, P-value threshold for pairwise assembly was 1e-9, for extension and refinement steps was 1e-10, and for final merging was 1e-11. The false positive parameter and false negative parameter were set to 1.5/100 Kb and 0.15. Finally, 2202 maps which spanned 2.91 Gb genome were assembled for *M. pentadactyla* and 2096 maps which spanned 2.85 Gb genome were assembled for *M. javanica*, with N50 length 1.884 Mb and 1.972 Mb respectively (Table 1). The largest fragment of *M. pentadactyla* is about 14.21 Mb in size with 1354 label sites, and the largest fragment of *M. javanica* is about 10.39 Mb in size with 1004 label sites (Fig. 2).

Whole-genome comparison between these two species were carried out by RefAligner with the P-value of 1e-9. The result show that 2196 maps covering 2.86 Gb from *M. pentadactyla* and 2088 maps covering 2.78 Gb from *M. javanica* can be mapped to each other with the map rate of 97.544% and 98.282%. Totally, 23,631 alignment blocks were generated. However, several reverse alignment were found in those blocks suggested a series of large genome rearrangements events during the divergence and evolution of these two species (Fig. 3).

Several phylogenetic investigation have been conducted in Manidae like using *SRY* gene,

COX I gene and whole mitochondria sequence [10]. Meanwhile, a series genome projects for pangolins are ongoing. But until now, no genome wide comparison have been reported. Here we represent two optical maps of *M. pentadactyla and M. javanica* using Irys system. These maps can be reliable reference for the further genome assembly. The comparison of those two maps reveal the similarity and difference between *M. pentadactyla and M. javanica*, showing the potential genome rearrangement events during Manidae evolution. Our work implies that optical mapping is a faithful long-range linkage information for genome assembly, which also can be a respectable choice for genome-wide highly related species comparison with convenience and low-cost.

Availability and requirements of software used:

IrysView 2.4 can be got from Bionano Genomics (http://bionanogenomics.com/products/irysview/). The software requires are Windows Python Runtime v2.7.5, Microsoft .Net 4.5.2, and Irys tools (RefAligner and Assembler, which can be found at http://bionanogenomics.com/support/software-updates/).

Availability of supporting data and materials:

Datasets which supporting this Data Note are deposited at the GigaScience repository, GigaDB [11].

Abbreviations

NGS: next-generation sequencing; HMW DNA: high molecular weight DNA; PBS: phosphate buffered saline; SV: structural variation.

Ethics approval

All animal studies were reviewed and approved by the animal ethics review committee of Guangdong Provincial Hospital of Chinese Medicine.

Consent for publication

Not applicable

Competing interests

Liao Baosheng and Zhai Chaochao are employees of Ultravision-tech.

Funding

This work is support by National Nature Science Foundation of China, the project number are 81403053 and 81503469.

Authors' contributions

CSL, HZH and XJ designed the project. HZH, XSM, GY prepared the samples. XJ, LBS, ZCC performed the experiments. LBS, XJ, XSM, HZH analyzed the data. CSL, XJ, HZH, LBS, QXH wrote the manuscripts.

Acknowledgements

We would like to thank Dr Chu Yang, Miss Bai Rui and Mr Ren Jian for the useful suggestions on data interpretation.

Authors' details

[1]Guangdong Provincial Hospital of Chinese Medicine, Guangzhou 510006, China. [2]Institute of Chinese Materia Medica, China Academy of Chinese Medicines, Beijing 100700, China. [3]Ultravision-tech, Beijing 100089, China. [4]Institute of Medicinal Plant Development, Chinese Academy of Medical Sciences, Beijing 100193, China

References

1. Gaudin TJ, Emry RJ, Wible JR. The phylogeny of living and extinct Pangolins (Mammalia, Pholidota) and associated taxa: A morphology based analysis. J Mamm Evol. 2009;16(4):235-305. doi:10.1007/s10914-009-9119-9.
2. Teo ASM, Verzotto D, Yao F, Niranjan N, Hillmer AM. Single-molecule optical genome mapping of a human HapMap and a colorectal cancer cell line. GigaScience. 2015;4(1):1-6. doi:10.1186/s13742-015-0106-1.
3. Tang H, Lyons E, Town CD. Optical mapping in plant comparative genomics. GigaScience. 2015;4(1):1-6. doi:10.1186/s13742-015-0044-y.
4. Lam ET, Hastie A, Lin C, Ehrlich D, Das SK, Austin MD, et al. Genome mapping on nanochannel arrays for structural variation analysis and sequence assembly. Nat Biotech. 2012;30(8):771-6.

doi:10.1038/nbt.2303.

5. Chamala S, Chanderbali AS, Der JP, Lan T, Walts B, Albert VA, et al. Assembly and validation of the genome of the nonmodel basal angiosperm *Amborella*. Science. 2013;342(6165):1516-7. doi:10.1126/science.1241130.

6. Dong Y, Xie M, Jiang Y, Xiao N, Du X, Zhang W, et al. Sequencing and automated whole-genome optical mapping of the genome of a domestic goat (*Capra hircus*). Nat Biotech. 2013;31(2):135-41. doi:10.1038/nbt.2478.

7. Vanburen R, Bryant D, Edger PP, Tang H, Burgess D, Challabathula D, et al. Single-molecule sequencing of the desiccation-tolerant grass *Oropetium thomaeum*. Nature. 2015;527(7579):508-11. doi:10.1038/nature15714.

8. Chen S, Xu J, Liu C, Zhu Y, Nelson DR, Zhou S, et al. Genome sequence of the model medicinal mushroom *Ganoderma lucidum*. Nat Commun. 2012;3(2):177-80. doi:10.1038/ncomms1923.

9. Grunwald A, Dahan M, Giesbertz A, Nilsson A, Nyberg LK, Weinhold E, et al. Bacteriophage strain typing by rapid single molecule analysis. Nucleic Acids Res. 2015;43(18). doi:10.1093/nar/gkv563.

10. Qin X, Dou S, Guan Q, Qin P, She Y. Complete mitochondrial genome of the *Manis pentadactyla* (Pholidota, Manidae): Comparison of *M. pentadactyla* and *M. tetradactyla*. Mitochondr DNA. 2012;23(1):37-8. doi:10.3109/19401736.2011.643881.

11.

Figure legend

Figure 1 Irys raw images

Labeled HMW DNA were linearized in nano channel. Restriction site were digest by Nt.BspQI and labeled by fluorescence dNTP. DNA backbone (1a, 1c) and labels (1c, 1d) were detected by EM CCD with blue (473 nm) and green (532 nm) laser. Fig.1a and 1b are raw images of *M. pentadactyla*; 1c and 1d are raw images of *M. javanica*. Raw molecules data were detected by Irys AutoDetect 2.1.4 from raw images.

Figure 2 Assembled physical map

Physical map were assembled and extended by the similarity and overlap of molecules. The blue bar stands for the physical map, the green bar for the molecule. In the absence of amplification, the molecule coverage of each part of physical map is very uniform. 2a and 2b are physical map examples of *M. pentadactyla* and *M. javanica* respectively.

Figure 3 Physical map comparison of *M. pentadactyla* and *M. javanica*

The physical maps of *M. pentadactyla* and *M. javanica* were compared by similarity. The physical map of *M. pentadactyla* was shown at the top, and *M. javanica* at the bottom. The line in the middle shows the links of similar regions. The comparison shows that the two species have high similarity in physical map level (more than 97% were aligned to each other). But there are some area were insertions/deletions, or inversion were found.

Table legend

Table 1 Statistics of physical map data of *M. pentadactyla* and *M. javanica*

Table 1

	M. pentadactyla	*M. javanica*
RawData		
Quantity (Gb)	517.874 (178X)	504.743 (177X)
Number of Molecules	3,313,734	3,439,885
Molecule N50 (Kb)	216.3	212.4
Label Density (/100Kb)	11.7	11.4
Label SNR	11.7	10.8
Filtered Data		
Molecule Length Threshold (Kb)	150	150
Label SNR Threshold	3	3
Quantity (Gb)	360.483 (123X)	339.814 (119X)
Number of Molecules	1,360,730	1,254,380
Molecule N50	275.5	281.1
Label Density	10.193	10.151
Label SNR	12.7	11.4
Assemble Statistics		
Total Length (Gb)	2.91	2.85
Number of Maps	2202	2094
Map N50 (Mb)	1.884	1.972
Average Map Length (Mb)	1.32	1.36
Max Map Length (Mb)	14.205	10.385

Pairwise Alignment		
Aligned Map number	2196	2088
Total Aligned Length (Mb)	4904.52	4716.849
Unique Aligned Length (Mb)	2861.22	2783.567

Figure 1 Click here to download Figure Fig. 1.png

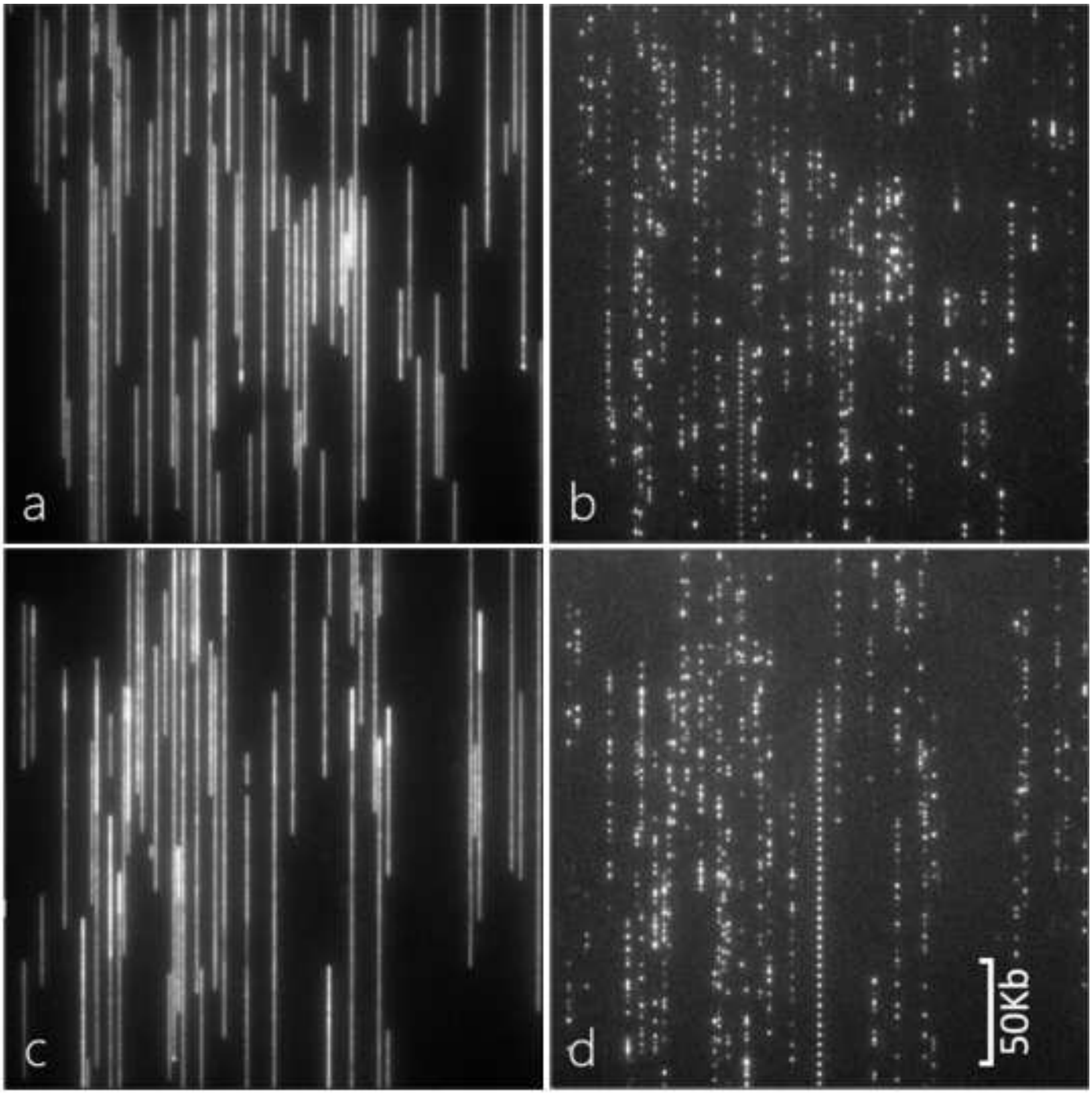

Figure 2

Click here to download Figure Fig. 2.png

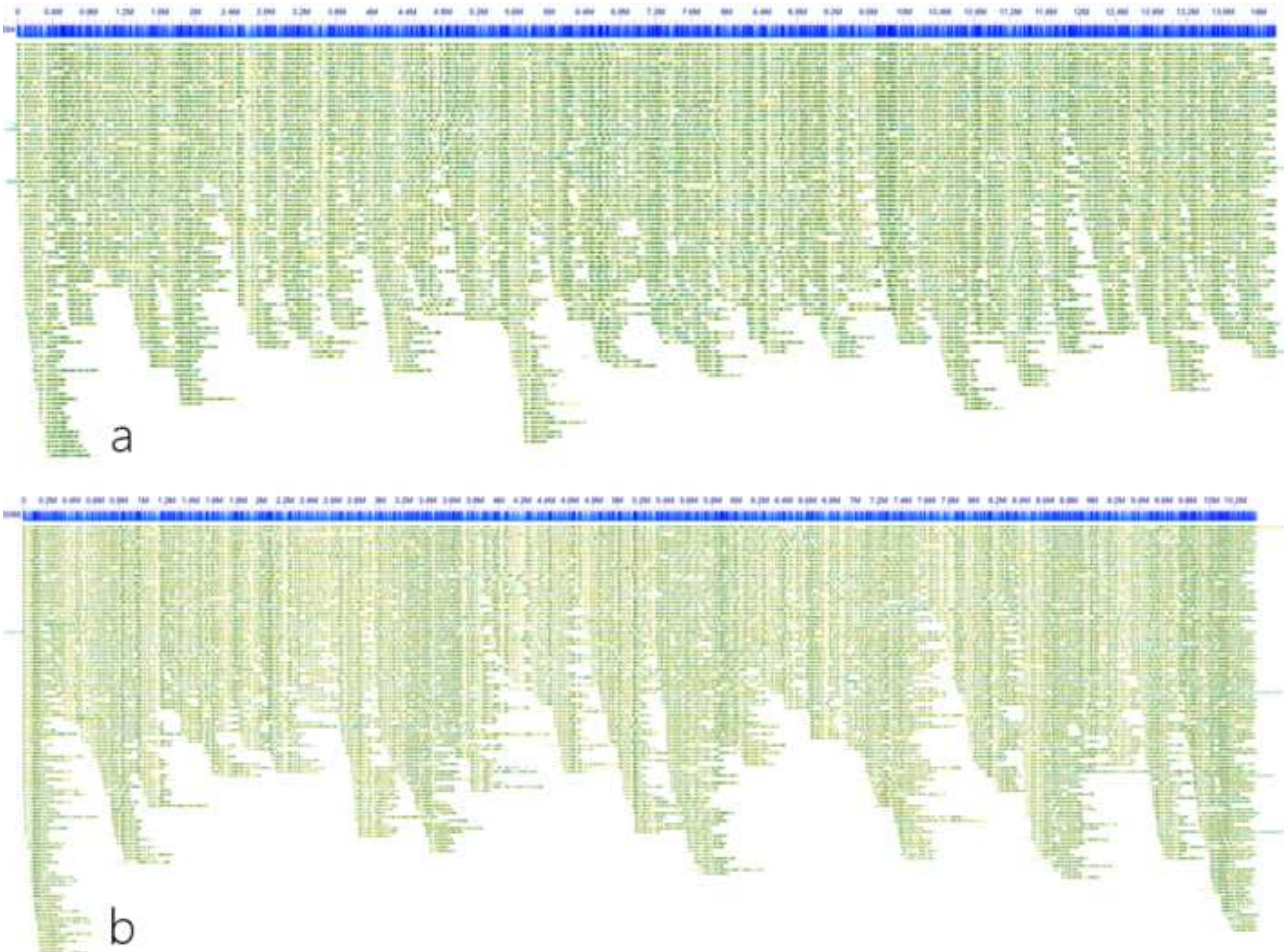

Figure 3

Click here to download Figure Fig. 3.png

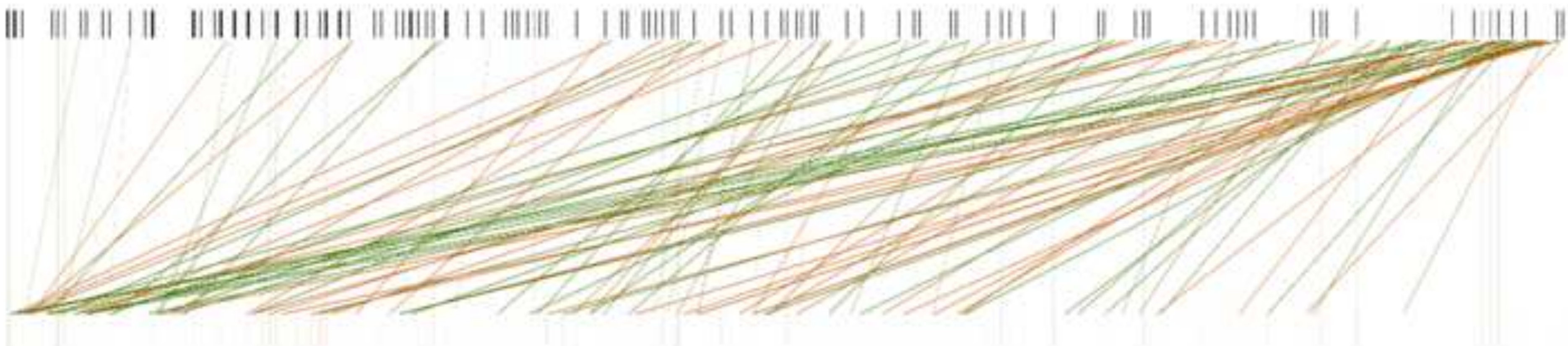

 Click here to download Personal Cover Cover letter.docx

Dear editor,

It’s a great honor for us to submit our manuscript entitled “Comparative optical genome analysis of two Pangolin species *Manis pentadactyla* and *Manis javanica*” for your consideration of publication as Data Note in *GigaSciecne*.

Optical mapping has been widely used for the improvement of genome assembly and for the long range structure variation analysis. So far, a large set of optical maps have been released such as those for Amborella, goat, *Oropetium thomaeum* and *Ganoderm lucidum*. However, most work just reckoned optical maps as an accessibility tool for NGS sequencing but ignoring its significant advantages in comparative genomics. As a tool for large molecules imaging, optical mapping offered a complementary sight for sequence variation especially for panoramic analysis of chromosome variation which is widespread during higher organisms diversifications. Manis is a group of particular mammals with scale covering their skin. Its unique evolutionary characteristics aroused zoologists’ interesting. Here, we presented the optical maps of *Manis pentadactyla* and *Manis javanica*, in order to reveal their genetic structure and to seek their interspecific variations. To our knowledge, this data set is the first report descripting segmental variation between vertebrate animals. This work will contribute important insights into the genome evolution of Manis.

We believe our data are solid and faithful and we deem the use of optical mapping as a comparative genomics tools will attracted the interesting of broader scientific audiences. We hope this manuscript will be considered suitable for publication in GigaScience, and we look forward to your feedback.

If any other questions or comments, please do not hesitate to contact me, thank you.

With kind regards,

Yours sincerely,

Huang Zhihai

Guangdong Provincial Hospital of Chinese Medicine,

Guangzhou 510006, China

Email: zhhuang7308@163.com
