# Supplementary material for: Comparative optical genome analysis of two pangolin species: Manis pentadactyla and Manis javanica
Source: Gigascience. 2016 Dec 29;5(1):1–5. doi: 10.1093/gigascience/giw001 (PMC6082621; doi:10.1093/gigascience/giw001)
Supplement: Supplementary file 2 [file giw001_giga-d-16-00053_revision-1.pdf]

Manuscript
Click here to view linked References
Click here to download Manuscript Main manuscript text R1.docx

Data Note

Title: Comparative optical genome analysis of two Pangolin species: *Manis pentadactyla* and *Manis javanica*

Authors: Huang Zhihai[1,*,&], Xu Jiang[2,&], Xiao Shuiming[2,&], Liao Baosheng[2,3,&], Gao Yuan[4], Zhai Chaochao[3], Qiu Xiaohui[1], Xu Wen[1], Chen Shilin[2,*]

1 Guangdong Provincial Hospital of Chinese Medicine, ; The Second Affiliated Hospital of Guangzhou University of Chinese Medicine; China Academy of Chinese Medical Sciences Guangdong Branch, China Academy of Chinese Medical Sciences, Guangzhou 510006, China

2 Institute of Chinese Materia Medica, China Academy of Chinese Medicines, Beijing 100700, China

3 Ultravision-tech, Beijing 100089, China

4 Institute of Medicinal Plant Development, Chinese Academy of Medical Sciences, Beijing 100193, China

* Correspondence: Huang Zhihai, zhhuang7308@163.com; Chen Shilin, slchen@icmm.ac.cn.

& Contributed to this paper equally.

Abstract:

Background: The ~~P~~pangolin is a Pholidota mammal with large keratin scales protecting its skin.~~,~~ ~~t~~Two pangolin species ~~of which~~ (*Manis pentadactyla* and *Manis javanica*) ~~-~~have been recorded as critically endangered on the International Union for Conservation of Nature ~~IUCN~~ Red List of Threatened Species. Optical mapping constructs high-resolution restriction maps from a single~~-stained~~-strand DNA molecule~~, then it allows the~~ for genome analysis ~~of genome at~~at the –mega-base scale ~~as well as the~~and to assist~~ance~~ ~~of~~ genome assembly. Here, we constructed ~~the~~ restriction maps of *M. pentadactyla* and *M. javanica* using optical mapping ~~hoping~~ to ~~help~~ assist with ~~these species~~ genome assembly and analysis of these species.

Findings: Genomic DNA was nicked with Nt.BspQI, followed by labeling ~~use~~ using ~~certain~~ fluorescent-~~-~~labeled bases~~, which~~ that were detected ~~in~~ by the Irys optical mapping system. ~~Totally,~~In total, 3,313,734 DNA molecules (517.847 Gb) for *M. pentadactyla* and 3,439,885 DNA molecules (504.743 Gb) for *M. javanica* were obtained, which corresponded~~s~~ to ~~about~~ approximately 178X and 177X genome coverage, respectively. Qualified molecules (≥150 Kb with a ~~and the~~ label density of > 6 sites/100 kilobases) were analyzed using the *de novo* assembly program embedded in the IrysView pipeline. We obtained two~~Two~~ maps that were ~~with~~ ~~genome size~~ 2.91 Gb and 2.85 Gb in size ~~were obtained, the maps~~with N50s of ~~are~~ 1.88 Mb and 1.97 Mb, respectively.

Conclusions: Optical mapping reveals~~ed~~ large-scale structural information that is especially important for ~~the~~ non-model genomes that ~~without~~ lack a good reference. ~~Further,~~The approach has ~~it holds~~ the potential ~~for the guidance~~to guide ~~of~~ NGS-based *de novo* assembly. Our data provide~~s~~ a resource for Manidae genome analysis and references for

de novo assembly. This note also~~ implys~~ provides ~~a ~~new insights ~~for ~~into Manidae evolutionary analysis at the genome ~~-~~structure level.

Key words: optical mapping, restriction maps, pangolin, Manidae

Data description:

Background

Pangolins are the~~,~~ sole representative~~ss~~ of ~~the ~~order Pholidota. Pangolins are~~,~~ ~~is ~~a group of nocturnal mammals that are well-known ~~by ~~for their full armor of scales. ~~Around the world,~~Only eight pangolin ~~- only contains eight ~~species exist worldwide.~~, and t~~These species have been distributed into three genera ~~-~~(Manis, Phataginus and Smutsia) according to Gaudin ~~*et al* ~~*et al.* [1]. *Manis pentadactyla* and *Manis javanica* belong to the Asian ~~subfamily of~~ Pangolin subfamily~~, these of which ~~ and ~~has~~have long been~~ve~~ ~~long been ~~used as an ingredient in traditional medicine in China and ~~S~~southeast~~outh-east~~ Asia~~ for a long time~~. ~~Due to poaching and deforestation, t~~Their coloniesy~~y~~ and habitats have been largely destroyed due to poaching and deforestation, and these two species are on the verge of extinction. ~~Now ~~Currently, *M. pentadactyla* and *M. javanica* ~~have been~~are recorded as critically endangered on the IUCN Red List of Threatened Species.

Optical mapping is a molecular tool for chromosome-wide restriction map~~s~~ production [2]. During the optical mapping process, stretched linear DNA ~~were ~~is labeled at specific sequence motifs and~~ves~~ then~~ were ~~ exposed under a fluorescence microscope~~ for~~ to generate an~~ing~~ image signal, which translates~~d~~ to motif-distance information for further analysis [~~3,~~ ~~4~~3]. ~~Unlike traditional sequencing approaches, o~~Optical mapping possesses several advantages over traditional sequencing approaches, such as single molecule analysis and long

DNA molecules, ~~which~~ and can be used for *de novo* map~~s~~ assembly or sequencing contig~~s~~ anchoring. ~~So far~~To date, optical mapping has~~ve~~ facilitated or improved ~~a~~ large ~~arrays of~~ genome assembly arrays,~~ies~~ including ~~Amborella [5], goat~~humans [~~6~~4, 5], *Oropetium thomaeum* [~~7~~6] and *Ganoderm lucidum* [~~8~~7]. ~~Beyond the guide of~~In addition to genome assembly guidance, optical mapping offers~~ed~~ a complementary ~~sight~~ approach for sequence variation analysis in large-region comparisons ~~instead of~~in addition to nucleotide matches~~,~~ ~~which~~ and provides~~s~~ several unique traits for evolutionary or functional analyses~~is~~. At present, d~~D~~irect comparisons between optical maps are usually conducted in microbes~~,~~ but ~~it has been~~ are lacking ~~so far~~ in large genomes, such as ~~like~~ animals or plants [~~9~~8]. Here, we present~~ative~~ two optical Manis maps, and we ~~of Manis, in order to~~ reveal and compare their genetic structures~~, we also~~ ~~and compare them~~ ~~by~~ using pairwise sequence alignments to ~~seek~~ identify their interspecific variations.

DNA extraction, labelling and data collection

High molecular weight DNA was isolated from *M. pentadactyla* and *M. javanica* blood samples. ~~To be specific,~~A total of 3 ml of blood ~~was~~ from an orbital sample~~d and~~ was anticoagulated ~~by~~ with EDTA and~~,~~ then shipped on ice. ~~For each 3 ml sample,~~A total of 9 ml of RBC lysis solution ~~were~~ was mixed with each 3 ml sample and rocked gently at room temperature for 10 min. The mixture ~~were~~ was spun at 2000 x g at 4 °C for 2 min, and~~,~~ ~~then~~ the supernatant ~~were~~ was discarded. The pellet was suspended in 3 ml of PBS buffer. After removing~~e~~ the insoluble particulates, the mixture ~~were~~ was spun again. The supernatant ~~were~~ was discarded and the pellet ~~were~~ was resuspended in 563 µl of refrigerated cell suspension buffer~~, the cell number in the mixture should be~~ at a density of ~0.5×10^7 cells/ml. For gel

casting,~~ per~~ 75 µl of resuspension~~ed~~ buffer was mixed with 25 µl of preheated 2% agarose, and~~,~~ the gels were solidified at 4 °C for 45 min. The ~~G~~gel~~ s~~ casts were immersed in 5 ml of lysis buffer (0.5 M EDTA, pH 9.5,~~;~~ ~~1 %~~1% lauroyl sarcosine, sodium salt, and 2 mg/ml~~;~~ proteinase K~~, 2 mg/ml~~) at 50 °C for 2 days for cell lysis. The cell-lysed gels were washed with 1X TE~~, and~~; then, the immobilized DNA ~~were~~ was recovered by melting the gels at 70 °C for 10 min, followed by incubation with GELase (Epicentre~~er~~, USA) at 43 °C for 45 min. The recovered DNA ~~were~~ was drop dialyzed against TE for 4 h using a 0.1-~~-~~µm membrane. The ~~D~~dialyzed DNA ~~were~~ was quantified and stored for nicking.

~~Before~~ Prior to molecular nicking, the DNA was equilibrated at room temperature for 30 min and gently mixed with wide bore tips. In a 10-~~-~~µl reaction system, 300 ng of equilibrated DNA ~~with~~ was added to 7 units of Nt.BspQI (NEB, USA) nickase and 1 µl of nicking buffer ~~were added~~ and mixed. The nicking process was conducted in a thermal cycler at 37 °C for 2 h. The nicked DNA was incubated ~~added~~ with 5 µl of labeling master mix ~~which~~ containing~~ed~~ 1.5 µl of labeling buffer (~~Bionano,~~BioNano Genomics, USA), 1.5 µl of labeling mix (~~Bionano,~~BioNano Genomics, USA), and 1 µl of Taq polymerase (NEB, USA) ~~for~~ to flag~~ging~~ ~~certain motivess~~specific motifs. The labeling process was conducted at 72 °C for 1 h. Each labeled DNA solution was mixed with 15 µl of Repair Master Mix ~~which~~ containing~~ed~~ 0.5 µl of 10 Thermo polymerase buffer (NEB, USA), 0.4 µl of 50X repair mix (~~Bionano,~~BioNano Genomics, USA), 0.4 µl of 50 mM NAD+ (NEB, USA), 1.0 µl of Taq DNA polymerase (NEB, USA), and 2.7 µl of ultrapure water for nick~~s~~ repair. The repair~~ing~~ reaction was conducted at 37 °C for 30 mins, followed~~ing~~ by the addition of 1 µl of stop solution (~~Bionano,~~BioNano Genomics, USA) to stop the reaction. After ligating~~ed~~ the nicks,

the backbone of the labelled DNA was stained with IrysPrep DNA stain solution (~~Bionano,~~BioNano Genomics, USA) at 4 °C overnight. The ~~P~~prepared samples were loaded onto Irys chips (~~Bionano,~~BioNano Genomics, USA) ~~then~~ and then applied to the chip ~~full filled the~~ nano-channels~~ in chip,~~. ~~t~~The concentration time was ~~set to~~ 400 s ~~in order~~ to avoid over-staining or over-loading. The ~~F~~fluorescent~~ly~~ ly –labelled DNA ~~were~~ was illuminated ~~by certain~~by the corresponding laser, and the signal was caught by an onboard EM CCD camera (Fig. 1). The acquired images were converted to digital data ~~by~~ with the Auto-detect software. ~~Totally~~In total, 517.874 Gb and 504.743 Gb of dat~~e~~a were generated for *M. pentadactyla* and *M. javanica*, represent~~ed~~ing 178X and 177X coverage of their predicted genomes, respectively.

Genome assembly

All ~~the~~ data were filtered by IrysView~~ under~~ using the following criteria: molecule lengths ≥150 k~~K~~b and~~,~~ a label signal/noise ratio (SNR) ≥ 3. The number of filtered molecules ~~is about~~was approximately 1,360,730 for *M. pentadactyla* with a N50 length of 275.5 k~~K~~b and 1,254,380 for *M. javanica* with a N50 length of 281.1 k~~K~~b. The label density~~ is~~ was 10.193/100 k~~K~~b for *M. pentadactyla* and 10.151/100 k~~K~~b for *M. javanica*. The distance between adjacent labels ranged from 0 k~~K~~b to 833.609 k~~K~~b for *M. pentadactyla* and 0 k~~K~~b to 955.352 k~~K~~b for *M. javanica*. ~~In the detecting~~During the detection process, two label sites ~~which are~~that are near ~~to~~ each other will be detected ~~as~~ because they ~~one or can't not~~cannot be separated~~, so~~; therefore, the distance between ~~of~~ these sites will be ~~set to~~ 0 bp. Simple tandem repeat areas~~,~~ ~~whose~~ with repeat units with only ~~has~~ one restriction site~~,~~ were detected by the molecules. The statistical analysis~~s~~ revealed that the most ~~of the appeared~~common simple tandem repeat unit size ~~is~~ was 4.3 k~~K~~b, ~~and the second~~followed by ~~abundant repeat~~

~~size is~~ 5.2 k~~K~~b in *M. pentadactyla*~~,~~ and 4.6 k~~K~~b and 3.4 k~~K~~b in *M. javanica*. The total length of the repeat region accounted for 0.55% and 0.45% of the raw data. The RefAligner and Assembler packages in IrysView were ~~invoked~~ used for *de novo* assembly. The assembly~~e~~ process comprised ~~by~~ a molecule~~s~~ pairwise comparison, graph building and map~~s~~ refinement. In this work, the P-value thresholds were 1e-9 for the pairwise assembly, ~~was 1e-9,~~ 1e-10 for the extension and refinement steps ~~was 1e-10~~, and 1e-11 for the final merging ~~was 1e-11~~. The false positive ~~parameter~~ and false negative parameters were set to 1.5/100 k~~K~~b and 0.15/100 kb. Finally, 2202 maps ~~which~~ spanning~~ed~~ 2.91 Gb of the genome were assembled for *M. pentadactyla* and 2096 maps ~~which~~ spanning~~ed~~ 2.85 Gb of the genome were assembled for *M. javanica*, with N50 lengths of 1.884 Mb and 1.972 Mb, ~~respectively (~~respectively (Table 1). The largest ~~fragment of~~ *M. pentadactyla* ~~is about~~fragment was approximately 14.21 Mb in size with 1354 label sites, and the largest ~~fragment of~~ *M. javanica* ~~is about~~fragment was approximately 10.39 Mb in size with 1004 label sites (Fig. 2).

Genomics comparison

A ~~W~~whole-genome comparison between these two species ~~were~~ was ~~carried out~~performed with ~~by~~ RefAligner ~~with the~~using a P-value of 1e-9. The results showed that 2196 maps covering 2.86 Gb from *M. pentadactyla* and 2088 maps covering 2.78 Gb from *M. javanica* ~~can be~~could be mapped to ~~each other~~one another with ~~the~~ map rates of 97.544% and 98.282%, respectively. ~~Totally~~In total, 23,631 alignment blocks were generated. However, several reverse alignments were found in ~~those~~ the blocks, suggesting~~ed~~ that a series of large genome rearrangement~~s~~ events occurred during the divergence and evolution of these two species (Fig. 3).

Conclusion

Several phylogenetic investigations have been conducted in Manidae ~~like~~ sequences using the *SRY* gene, *COX I* gene and whole mitochondrial sequence [~~10~~9]. ~~Meanwhile, a~~ A series of genome projects for pangolins ~~are~~ is ongoing. ~~But until now~~However, no genome-wide comparisons have been reported to date. Here, we ~~re~~present two optical maps of *M. pentadactyla* and *M. javanica* generated using the Irys system. These maps can ~~be~~ serve as reliable references for ~~the~~ further genome assembly. The comparison of the~~ose~~ two maps revealed ~~the~~ similarities~~y~~ and differences between *M. pentadactyla* and *M. javanica* and~~,~~ showed~~ing~~ that ~~the~~ potential genome rearrangement events occurred during Manidae evolution. Our work implies that optical mapping ~~is~~ provides ~~a faithful~~reliable long-range linkage information for genome assembly~~, which also~~ and can~~-~~ be a ~~respectable~~ suitable choice for convenient and low-coast genome-wide comparisons of highly related species ~~comparison with convenience and low-cost~~.

Availability and software requirements ~~of software used~~:

IrysView 2.4 can be ~~got~~ obtained from BioN~~n~~ano Genomics (http://bionanogenomics.com/products/irysview/). The software requirements are as follows: Windows Python Runtime v2.7.5, Microsoft .Net 4.5.2, and Irys tools (RefAligner and Assembler, which can be found at http://bionanogenomics.com/support/software-updates/).

Availability of supporting data and materials:

Datasets ~~which~~ supporting this Data Note are deposited at the GigaScience repository,

GigaDB [~~11~~10].

Abbreviations

NGS: next-generation sequencing; HMW DNA: high molecular weight DNA; PBS: phosphate buffered saline; SV: structural variation.

Ethics approval

All animal studies were reviewed and approved by the animal ethics review committee of Guangdong Provincial Hospital of Chinese Medicine.

Consent for publication

Not applicable

Competing interests

Liao Baosheng and Zhai Chaochao are employees of Ultravision-tech.

Funding

This work is supported by the National Nature Science Foundation of China~~, the under project numbers are~~ (81403053 and 81503469), Guangdong Provincial Hospital of Chinese Medicine Special Fund (2015KT1817) and China Academy of Chinese Medical Sciences Secial Fund for Health Service Development of Chinese Medicine.

Authors' contributions

CSL, HZH and XJ designed the project. HZH, XSM, and GY prepared the samples. XJ, LBS, and ZCC performed the experiments. LBS, XJ, XSM, and HZH analyzed the data. CSL, XJ, HZH, LBS, and QXH wrote the manuscript~~s~~.

Acknowledgements

We ~~would like to~~ thank Dr. Chu Yang, Miss Bai Rui and Mr. Ren Jian for ~~the~~ useful suggestions on data interpretation.

Authors' details

[1]Guangdong Provincial Hospital of Chinese Medicine~~,~~; The Second Affiliated Hospital of Guangzhou University of Chinese Medicine; China Academy of Chinese Medical Sciences Guangdong Branch, China Academy of Chinese Medical Sciences, Guangzhou 510006, China. [2]Institute of Chinese Materia Medica, China Academy of Chinese Medicines, Beijing 100700, China. [3]Ultravision-tech, Beijing 100089, China. [4]Institute of Medicinal Plant Development, Chinese Academy of Medical Sciences, Beijing 100193, China

References

1. Gaudin TJ, Emry RJ, Wible JR. The phylogeny of living and extinct Pangolins (Mammalia, Pholidota) and associated taxa: A morphology based analysis. J Mamm Evol. 2009;16(4):235-305. doi:10.1007/s10914-009-9119-9.

2. Teo ASM, Verzotto D, Yao F, Niranjan N, Hillmer AM. Single-molecule optical genome mapping of a

human HapMap and a colorectal cancer cell line. GigaScience. 2015;4(1):1-6. doi:10.1186/s13742-015-0106-1.

~~3. Tang H, Lyons E, Town CD. Optical mapping in plant comparative genomics. GigaScience. 2015;4(1):1-6. doi:10.1186/s13742-015-0044-y.~~

~~4~~3. Lam ET, Hastie A, Lin C, Ehrlich D, Das SK, Austin MD, et al. Genome mapping on nanochannel arrays for structural variation analysis and sequence assembly. Nat Biotech. 2012;30(8):771-6. doi:10.1038/nbt.2303.

~~5~~4. Pendleton M, Sebra R, Pang AW, Ummat A, Franzen O, Rausch T, et al. Assembly and diploid architecture of an individual human genome via single-molecule technologies. Nature Methods. 2015;12(8):780-6. doi:10.1038/nmeth.3454.~~Chamala S, Chanderbali AS, Der JP, Lan T, Walts B, Albert VA, et al. Assembly and validation of the genome of the nonmodel basal angiosperm *Amborella*. Science. 2013;342(6165):1516-7. doi:10.1126/science.1241130.~~

~~6~~5. Mostovoy Y, Levy-Sakin M, Lam J, Lam ET, Hastie AR, Marks P, et al. A hybrid approach for *de novo* human genome sequence assembly and phasing. Nature Methods. 2016;13(7):587-90. doi:10.1038/nmeth.3865.~~Dong Y, Xie M, Jiang Y, Xiao N, Du X, Zhang W, et al. Sequencing and automated whole-genome optical mapping of the genome of a domestic goat (*Capra hircus*). Nat Biotech. 2013;31(2):135-41. doi:10.1038/nbt.2478.~~

~~7~~6. Vanburen R, Bryant D, Edger PP, Tang H, Burgess D, Challabathula D, et al. Single-molecule sequencing of the desiccation-tolerant grass *Oropetium thomaeum.* Nature. 2015;527(7579):508-11. doi:10.1038/nature15714.

~~8~~7. Chen S, Xu J, Liu C, Zhu Y, Nelson DR, Zhou S, et al. Genome sequence of the model medicinal mushroom *Ganoderma lucidum.* Nat Commun. 2012;3(2):177-80. doi:10.1038/ncomms1923.

~~9~~8. Grunwald A, Dahan M, Giesbertz A, Nilsson A, Nyberg LK, Weinhold E, et al. Bacteriophage strain typing by rapid single molecule analysis. Nucleic Acids Res. 2015;43(18). doi:10.1093/nar/gkv563.

~~10~~9. Qin X, Dou S, Guan Q, Qin P, She Y. Complete mitochondrial genome of the *Manis pentadactyla* (Pholidota, Manidae): Comparison of *M. pentadactyla* and *M. tetradactyla*. Mitochondr DNA. 2012;23(1):37-8. doi:10.3109/19401736.2011.643881.

~~11~~10. Huang Z, Xu J, Xiao S, Liao B, Gao Y, Zhai C, et al. Supporting data for "Comparative optical genome analysis of two Pangolin species: *Manis pentadactyla* and *Manis javanica*". GigaScience Database. 2016. http://

Formatted: EndNote Bibliography, Indent: Left: 0", Hanging: 0.5"

Formatted: Font: 10 pt, Check spelling and grammar

Figure legends

Figure 1 Irys raw images

Labeled HMW DNA ~~were~~ was linearized in the nano channel. The ~~R~~restriction sites were digested ~~by~~ with Nt.BspQI and labeled ~~by~~ with fluorescence dNTP. The DNA backbone (1a, 1c) and labels (1c, 1d) were detected by EM CCD with blue (473 nm) and green (532 nm) lasers. Fig. 1a and 1b are raw images ~~of~~ from *M. pentadactyla*; 1c and 1d are raw images ~~of~~ from *M. javanica*. The ~~R~~raw molecule~~s~~ data were detected ~~by~~ with Irys AutoDetect 2.1.4 from the raw images.

Figure 2 Assembled physical map

The ~~P~~physical maps were assembled and extended ~~by~~ based on the similarity and overlap of molecules. The blue bar ~~stands for~~indicates the physical map and~~,~~ the green bar ~~for~~ indicates the molecule. In the absence of amplification, the molecule coverage of each part of the physical map is very uniform. 2a and 2b are physical map examples of *M. pentadactyla* and *M. javanica*, respectively.

Figure 3 Physical map comparison of *M. pentadactyla* and *M. javanica*

The physical maps of *M. pentadactyla* and *M. javanica* were compared ~~by~~ based on similarity.

The physical map of *M. pentadactyla* ~~was~~ is shown at the top~~,~~ and *M. javanica* is shown at the bottom. The line in the middle shows the links ~~of~~ between similar regions. The comparison shows that the two species have high similarity ~~in~~ at the physical map level (~~more~~ greater than 97% ~~were~~ aligned to each other). ~~But there are~~However, some areas ~~can be found~~ ~~which~~contained ~~were~~ insertions/deletions~~,~~ or inversions ~~were found~~.

Table legend

Table 1 Statistical analysis~~s~~ of the ~~physical map data of~~ *M. pentadactyla* and *M. javanica* physical map data

Table 1

		M. pentadactyla	*M. javanica*
Raw Data			
	Quantity (Gb)	517.874 (178X)	504.743 (177X)
	Number of Molecules	3,313,734	3,439,885
	Molecule N50 (Kb)	216.3	212.4
	Label Density (/100Kb)	11.7	11.4
	Label SNR	11.7	10.8
Filtered Data			
	Molecule Length Threshold (Kb)	150	150
	Label SNR Threshold	3	3
	Quantity (Gb)	360.483 (123X)	339.814 (119X)
	Number of Molecules	1,360,730	1,254,380
	Molecule N50	275.5	281.1

Label Density	10.193	10.151
Label SNR	12.7	11.4
Assemblye Statistics		
Total Length (Gb)	2.91	2.85
Number of Maps	2202	2094
Map N50 (Mb)	1.884	1.972
Average Map Length (Mb)	1.32	1.36
Max Map Length (Mb)	14.205	10.385
Pairwise Alignment		
Aligned Map number	2196	2088
Total Aligned Length (Mb)	4904.52	4716.849
Unique Aligned Length (Mb)	2861.22	2783.567

Figure 1

Click here to download Figure Fig. 1.png

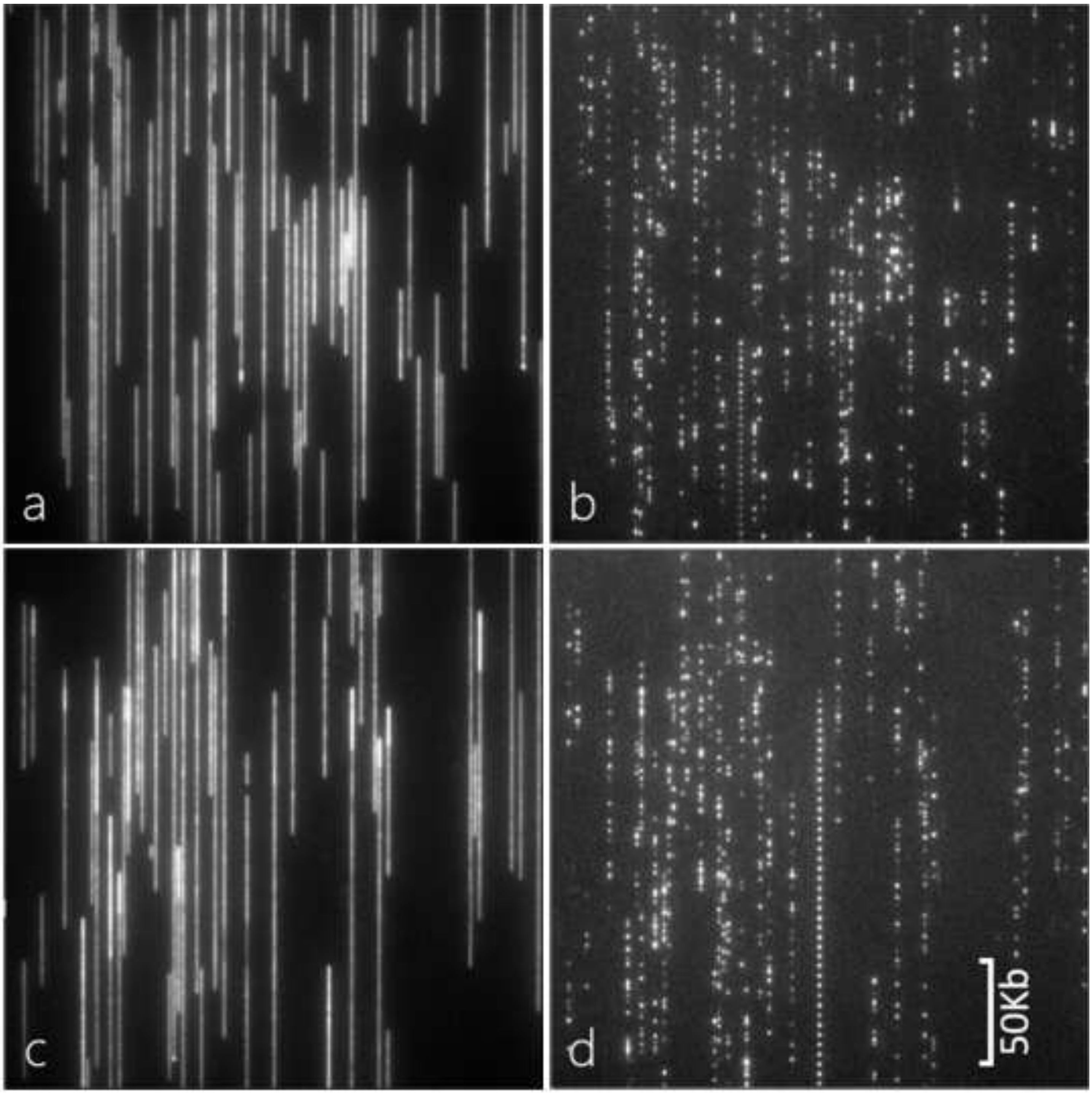

Figure 2

Click here to download Figure Fig. 2.png

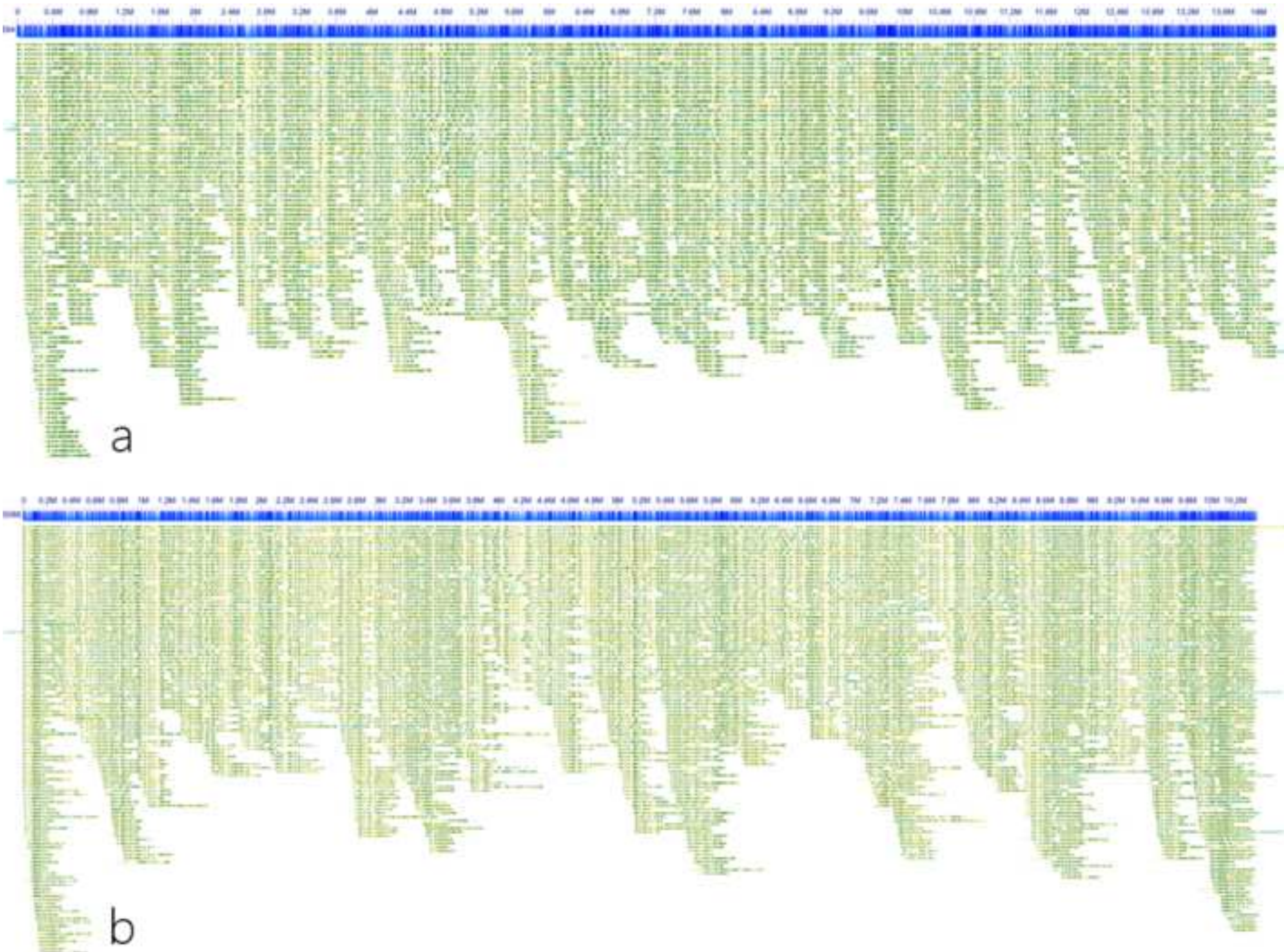

Figure 3

Click here to download Figure Fig. 3.tif

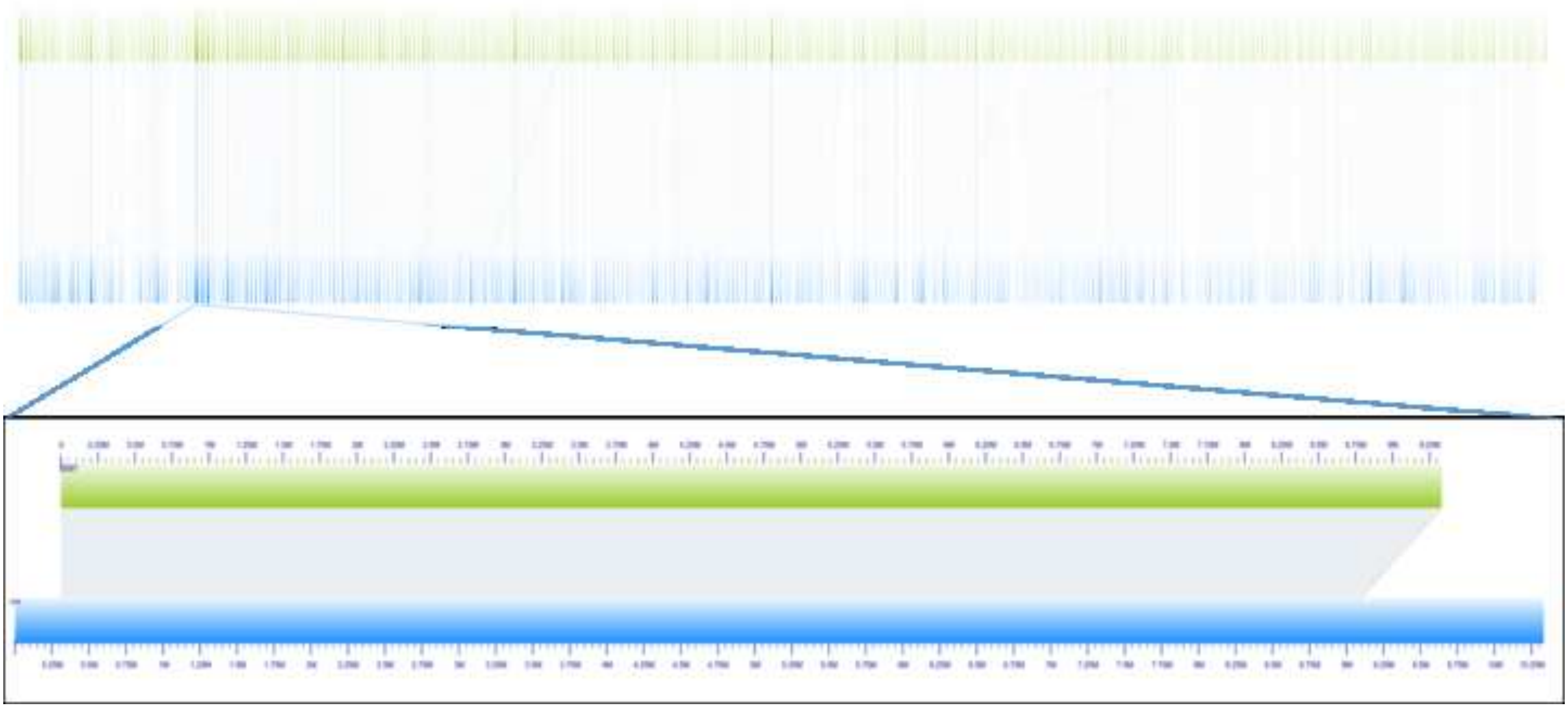

Dear Dr. Nicole Nogoy,

We are grateful for the detailed comments from you and the reviewers. We have carefully considered all of the comments and revised our manuscript accordingly. Afterward, we have sent our manuscript to American Journal Experts for further language editing. Below please find point-by-point replies to the comments and detailed explanations of all changes (V1 indicates the originally submitted version and R1 indicates the revised version; all revisions were tracked in R1).

Reviewer #1: Huang et al. described generation and availability of genome mapping data for two endangered pangolin species. Genome mapping is a relatively new physical mapping platform useful for genome assembly/finishing and structural variation analysis. The datasets would likely be of interest to researchers studying pangolins and potentially those interested in applying genome mapping to large genomes. However, discussion of validation of the datasets was limited.

We thank reviewer #1 for the positive comments.

Comments:

1. Why was it of interest to compare these two particular pangolin species? Are there key phenotypic differences between them? What is known about their evolutionary relationship to each other?

Reply: *Manis pentadactyla* and *Manis javanica* both belong to order Pholidota and genus *Manis* and have been listed as endangered species in the IUCN Red list. Pangolins are the only mammals with large protective keratin scales covering their skin. Pangolins are nocturnal with poor vision and capture food using their slender and soft tongues. Although they belong to the same genus and live in similar areas, there are several morphological differences between these two species. First, the ratio of the length of the middle claws of the hind feet and fore feet in *M. pentadactyla* is less than 0.5, whereas the ratio in *M. javanica* is greater than 0.5. Second, the length of the protruding rim of the external ear in *M. pentadactyla* is greater than 10 mm, whereas the length in *M. javanica* is less than 10 mm. Third, the number of single flank scales of the edge tail in *M. pentadactyla* is less than 21, whereas the number in *M. javanica* exceeds 21. However, no significant differences in body weight and the length of the hind feet were detected between these two species (Wu, *et al*, Acta Theriologica Sinica, 2004). A phylogenetic analysis using the mitochondrial genomes revealed that *M. pentadactyla* and *M. javanica* were the most closely related pangolin species (Hassanin, *et al*, Comptes Rendus Biologies, 2015). In East Asia, *M. pentadactyla* and *M. javanica* are used in traditional medicines, but we do not think that these different species have the exact same uses. Thus, we wanted to compare these two species due to their special evolutionary statuses and potential medicinal uses.

2. What other sequence/genetic datasets are publically available for these two and related species?

Reply: At the time we submitted our manuscript, no published genome data were available. A genome research article about these two species was released online on the 30th of August (Choo, *et al*, Genome Research, 2016), but the scaffold N50 values in this study (approximatey200 kb) were not desirable for chromosome-wide comparisons.

3. If the information is available, please comment on the genetic heterogeneity of thesamples. Were these animals from the wild or in captivity?

Reply: Our samples are in captivity. All of our operations followed the Ethics Committee Orientation of Guangdong Provincial Hospital of Chinese Medicine. The pangolins were housed in Qingfeng Park Medicinal Animal Research Institute, Dongguan, Guangdong Province, which complies with the Domestication and Breeding License of the Wildlife under Special State Protection authorized by the Forestry Administration of Guangdong Province. From Choo's research, the genetic heterogeneity of *M. javanica* is 0.15% and the genetic heterogeneity of *M. pentadactyla* is 0.04% (Choo, *et al*, Genome Research, 2016).

4. The authors cited both studies that featured traditional optical mapping and ones that featured BioNano Genomics' genome mapping platform. It might be confusing for researchers unfamiliar with optical mapping technologies.

Reply: Thank you for noting this problem. We have specified the optical mapping platform that we used in the current study and updated the references as follows:
(1) Reference 3 in V1: "Tang H, Lyons E, Town CD. Optical mapping in plant comparative genomics. GigaScience. 2015;4(1):1-6. doi:10.1186/s13742-015-0044-y." was deleted.
(2) Reference 4 in R1: "Pendleton M, Sebra R, Pang AW, Ummat A, Franzen O, Rausch T, et al. Assembly and diploid architecture of an individual human genome via single-molecule technologies. Nature Methods. 2015;12(8):780-6. doi:10.1038/nmeth.3454." replaced reference 5 in V1: "Chamala S, Chanderbali AS, Der JP, Lan T, Walts B, Albert VA, et al. Assembly and validation of the genome of the nonmodel basal angiosperm Amborella. Science. 2013;342(6165):1516-7. doi:10.1126/science.1241130."
(3) Reference 5 in R1: "Mostovoy Y, Levy-Sakin M, Lam J, Lam ET, Hastie AR, Marks P, et al. A hybrid approach for de novo human genome sequence assembly and phasing. Nature Methods. 2016;13(7):587-90. doi:10.1038/nmeth.3865." replaced reference 6 in V1: "Dong Y, Xie M, Jiang Y, Xiao N, Du X, Zhang W, et al. Sequencing and automated whole-genome optical mapping of the genome of a domestic goat (*Capra hircus*). Nat Biotech. 2013;31(2):135-41.

doi:10.1038/nbt.2478."

5. The authors claimed that the maps would be reliable references for further analyses. However, there is generally little information or evidence on how reliable these datasets might be. It would be helpful to discuss whether the observed data was consistent with expectation.

   5a. The authors mentioned that they collected 178X and 177X for the two species. What were the estimated genome sizes, and how were they determined? Were the assembly sizes close to the estimated genome sizes?

Reply: We calculated the data depth using the assembled physical map size. The size of the genome map generated from the BioNano Irys system was very close to the genome size estimated by sequence data or flow cytometry (the size of the genome map assembled from the BioNano Irys data was approximately 97% of the NGS assembly size (Vanburen, *et al*, Nature, 2015; Xiao, *et al*, BMC Genomics, 2015; Pendleton, *et al*, Nature Methods, 2015)). The genome map sizes in this study were 2.91 Gb (*M. pentadactyla*) and 2.85 Gb (*M. javanica*), which were comparable to Choo's research (Choo, *et al*, Genome Research, 2016).

   5b. The authors mentioned that the distance between adjacent labels could be up to ~833 kb, even though the average label density was ~10 labels per 100 kb. Does this correspond to the centromere or known repetitive regions in the genome?

Reply: Thank you for your careful and thoughtful question. These areas may be centromeres or highly repetitive regions. Due to the uneven distribution of restriction sites, these areas may also be areas with no restriction sites. At present, we cannot confirm the locations of the specific regions because we do not have a high quality reference for comparison.

6. Part of Figure 3 seemed to be missing. Also, based on the figure, the two species (at least visually) seemed quite different, while the author claimed that the two species had high similarity. The author might consider updating the figure or explaining how one would interpret the alignment and the apparent discrepancy.

Reply: We apologize for the confusion caused by this figure. The alignment in Figure 3 from V1 has not been sorted. We sorted the alignment based on the alignment order and added one alignment example in this figure, which represented an approximately 10 Mb-length alignment between two similar regions of *M. pentadactyla* and *M. javanica*. The green bars in Figure 3 indicate the genome maps of *M. pentadactyla* and the blue bars indicate the genome maps of *M. javanica*.

7. The authors claimed that optical mapping was convenient and low-cost. Please

briefly justify the statement.

Reply: A 200X sequencing depth is needed for any genome with an approximately 3 Gb nucleotide sequence, including shotgun libraries, mate-pair libraries and any other sequencing data from fosmids, BACs or linkage maps. The costs are approximately $20K for sequencing and $10K for library construction (only for shotgun and mate-pair, not including fosmids, BACs or linkage maps). Based on the species, the durations can range from 6 weeks to several months. In this study, only 3 chips were used for each species, which cost approximately $4.5K, and the whole process from DNA isolation to data analysis took only one week, which was obviously less than conventional NGS sequencing.

8. I would recommend using subheadings to separate the sections of the text under "Data description".

Reply: Thank you for the suggestion. We have added subheadings to separate the sections of the text under "Data description" to make the structure more explicit.
Paragraph 1-2: Background
Paragraph 3-4: DNA extraction, labelling and data collection
Paragraph 5: Genome assembly
Paragraph 6: Genomics comparison
Paragraph 7: Conclusion

9. I was not able to open the readme.txt file from the ftp site.

Reply: We apologize for the inconvenience. The readme.txt file contains brief descriptions of the uploaded files. The readme.txt file is attached here:

These data are associated with the manuscript "Comparative optical genome analysis of two Pangolin species: *Manis pentadactyla* and *Manis javanica*".

Authors:
Huang Zhihai[1,*,&], Xu Jiang[2,&], Xiao Shuiming[2,&], Liao Baosheng[2,3,&], Gao Yuan[4], Zhai Chaochao[3], Qiu Xiaohui[1], Chen Shilin[2,*]
Abstract:
Background: The pangolin is a Pholidota mammal with large keratin scales protecting its skin. Two pangolin species (*Manis pentadactyla* and *Manis javanica*) have been recorded as critically endangered on the IUCN Red List of Threatened Species. Optical mapping constructs high-resolution restriction maps from a single-strand DNA molecule for genome analysis at the mega-base scale and to assist genome assembly. Here, we constructed restriction maps of *M. pentadactyla* and *M. javanica* using optical mapping to assist with genome assembly and analysis of these species.
Findings: Genomic DNA was nicked with Nt.BspQI, followed by labeling using

fluorescent-labeled bases that were detected by the Irys optical mapping system. In total, 3,313,734 DNA molecules (517.847 Gb) for *M. pentadactyla* and 3,439,885 DNA molecules (504.743 Gb) for *M. javanica* were obtained, which corresponded to approximately 178X and 177X genome coverage, respectively. Qualified molecules (≥150 Kb with a label density of > 6 sites/100 kilobases) were analyzed using the *de novo* assembly program embedded in the IrysView pipeline. We obtained two maps that were 2.91 Gb and 2.85 Gb in size with N50s of 1.88 Mb and 1.97 Mb, respectively.
Conclusions: Optical mapping reveals large-scale structural information that is especially important for non-model genomes that lack a good reference. The approach has the potential to guide NGS-based *de novo* assembly. Our data provide a resource for Manidae genome analysis and references for *de novo* assembly. This note also provides new insights into Manidae evolutionary analysis at the genome structure level.

Data Description:
1. readme.txt: this introduction file
2. *M.pentadactyla*_RawMolecules.bnx: raw molecule data from *M. pentadactyla* generated from the BioNano Irys system
3. *M.javanica*_RawMolecules.bnx: raw molecule data from *M. javanica* generated from the BioNano Irys system
4. *M.pentadactyla*.cmap: physical map of *M. pentadactyla* assembled from raw molecules using the IrysSolve pipeline
5. *M.javanica*.cmap: physical map of *M. javanica* assembled from raw molecules using the IrysSolve pipeline

The data processing and data format instructions can be found at http://bionanogenomics.com/support/training/.

10. Under "Availability of supporting data and materials", it might be helpful to list what was deposited. Also, brief descriptions of the files would be helpful.

Reply: Thank you for your kind suggestion. We have included the data/file descriptions in the readme.txt.

11. There were grammatical issues; careful proofreading will be much needed.

Reply: Many thanks for the suggestion. We are sorry for our in-natural language. We have sent this version for language editing by native English speakers.

Reviewer #2: The Data Note by Zhihai et al. reports on construction of optical maps of two endangered species: *Manis pentadactyla* and *Manis javanica*. The authors

isolated DNA from blood of the two species and analysed them using Bionano Irys instrument. Using standard software IrysView software they constructed optical maps of the species and performed their pairwise alignment.

The paper summarizes information on this dataset: statistics on data generated, assembled maps, alignment and common repeat sizes are reported. Figures represent screenshots from Irys software.

The reported dataset will be complimentary to NGS genomics data, that is being generated for these ogranisms. It can assist de novo genome assembly of Manis species or improving NSG-based Manis assembly with new optical mapping.

The manuscript needs extensive editing and improvement of English language, it is difficult to read. For example:
Abstract, line 27: "high-solution restriction maps" -> "high-REsolution restriction maps"
Abstract, line 31: "followed by labeling use certain fluorescent labeled bases" -> "followed by labeling usING certain fluorescentLY labeled bases"
Abstract, line 34: "which about 178X" -> "which CORRESPONDS TO about 178x"
Abstract, line 39: "especially for the non-model genome that without good reference" ->"especially important for non-model genome that lacks a good reference"
... and so on throughout the whole manuscript. Please improve.

Reply: Thank you for the comments and for noting the language problem. We have corrected our expression. We are sorry for our in-natural language. This version have been sent for language editing by native English speakers.
