# Supplementary material for: Comparative optical genome analysis of two pangolin species: Manis pentadactyla and Manis javanica
Source: Gigascience. 2016 Dec 29;5(1):1–5. doi: 10.1093/gigascience/giw001 (PMC6082621; doi:10.1093/gigascience/giw001)

Manuscript
Click here to download Manuscript GIGA-D-16-00053 manuscript Cofactor edited 2-R2.docx

Data Note

Comparative optical genome analysis of two pangolin species: *Manis pentadactyla* and *Manis javanica*

Huang Zhihai[1*†], Xu Jiang[2†], Xiao Shuiming[2†], Liao Baosheng[1,2†], Gao Yuan[3], Zhai Chaochao[4], Qiu Xiaohui[1], Xu Wen[1] and Chen Shilin[2*]

[1]Guangdong Provincial Hospital of Chinese Medicine, The Second Affiliated Hospital of Guangzhou University of Chinese Medicine, and China Academy of Chinese Medical Sciences Guangdong Branch, China Academy of Chinese Medical Sciences, Guangzhou 510006, China

[2]Institute of Chinese Materia Medica, China Academy of Chinese Medicines, Beijing 100700, China

[3]Institute of Medicinal Plant Development, Chinese Academy of Medical Sciences, Beijing 100193, China

[4]Ultravision-tech, Beijing 100089, China

* Correspondence: Huang Zhihai, zhhuang7308@163.com; Chen Shilin, slchen@icmm.ac.cn

† Contributed equally

Abstract

Background: The pangolin is a Pholidota mammal with large keratin scales protecting its skin. Two pangolin species (*Manis pentadactyla* and *Manis javanica*) have been recorded as critically endangered on the International Union for Conservation of Nature Red List of Threatened Species. Optical mapping constructs high-resolution restriction maps from single DNA molecules for genome analysis at the megabase scale and to assist genome assembly. Here, we constructed restriction maps of *M. pentadactyla* and *M. javanica* using optical mapping to assist with genome assembly and analysis of these species.

Findings: Genomic DNA was nicked with Nt.BspQI and then labeled using fluorescently labeled bases that were detected by the Irys optical mapping system. In total, 3,313,734 DNA molecules (517.847 Gb) for *M. pentadactyla* and 3,439,885 DNA molecules (504.743 Gb) for *M. javanica* were obtained, which corresponded to approximately 178X and 177X genome coverage, respectively. Qualified molecules (≥150 kb with a label density of > 6 sites per 100 kb) were analyzed using the *de novo* assembly program embedded in the IrysView pipeline. We obtained two maps that were 2.91 Gb and 2.85 Gb in size with N50s of 1.88 Mb and 1.97 Mb, respectively.

Conclusions: Optical mapping reveals large-scale structural information that is especially important for non-model genomes that lack a good reference. The approach has the potential to guide *de novo* assembly of genomes sequenced using next-generation sequencing. Our data provide a resource for Manidae genome analysis and references for *de novo* assembly. This note also provides new insights into Manidae evolutionary analysis at the genome structure level.

Keywords: optical mapping, restriction maps, pangolin, Manidae

Data description {1st level heading}

Background {2nd level heading}

Pangolins are the sole representatives of order Pholidota. They are a group of nocturnal mammals that are well-known for their full armor of scales. Only eight pangolin species exist worldwide, which have been distributed into three genera (Manis, Phataginus and Smutsia) according to Gaudin et al. [1]. *Manis pentadactyla* and *Manis javanica* belong to the Asian Pangolin subfamily and have long been used as an ingredient in traditional medicine in China and southeast Asia, and their colonies and habitats have thus largely been destroyed by poaching and deforestation. These two species are on the verge of extinction; they are currently recorded as critically endangered on the International Union for Conservation of Nature Red List of Threatened Species.

Optical mapping is a molecular tool for chromosome-wide restriction map production [2]. During the optical mapping process, stretched linear DNA is labeled at specific sequence motifs and then exposed under a fluorescence microscope to generate an image signal, which translates to motif–distance information for further analysis [3]. Optical mapping has several advantages over traditional sequencing approaches, such as single molecule analysis and long DNA molecules, and can be used for *de novo* map assembly or sequencing contig anchoring. To date, optical mapping has facilitated or improved assembly arrays of large genomes, including humans [4, 5], *Oropetium thomaeum* [6], and *Ganoderm lucidum* [7]. In addition to genome assembly guidance, optical mapping offers a complementary approach for sequence variation analysis in large-region comparisons in

addition to nucleotide matches and provides unique traits for evolutionary or functional analyses. At present, direct comparisons between optical maps are usually conducted in microbes but are lacking in large genomes, such as animals or plants [8]. Here, we present two optical *Manis* maps, and we reveal and compare their genetic structures using pairwise sequence alignments to identify their interspecific variations.

DNA extraction, labeling and data collection {2nd level heading}

All animal studies were reviewed and approved by the animal ethics review committee of Guangdong Provincial Hospital of Chinese Medicine. High molecular weight DNA was isolated from *M. pentadactyla* and *M. javanica* blood samples. A total of 3 ml of blood from an orbital sample was anticoagulated with EDTA and then shipped on ice. A total of 9 ml of red blood cell lysis solution was mixed with each 3 ml sample and rocked gently at room temperature for 10 min. The mixture was spun at 2000 × *g* at 4 °C for 2 min, and the supernatant was discarded. The pellet was suspended in 3 ml of phosphate-buffered saline. After removing the insoluble particulates, the mixture was spun again. The supernatant was discarded and the pellet was resuspended in 563 μl of refrigerated cell suspension buffer at a density of ~0.5×10^7 cells/ml. For gel casting, 75 μl of resuspension buffer was mixed with 25 μl of preheated 2% agarose, and the gels were solidified at 4 °C for 45 min. The gel casts were immersed in 5 ml of lysis buffer (0.5 M EDTA, pH 9.5, 1% lauroyl sarcosine, sodium salt, and 2 mg/ml proteinase K) at 50 °C for 2 days for cell lysis. The cell-lysed gels were washed with 1× Tris-EDTA; then, the immobilized DNA was recovered by melting the gels at 70 °C for 10 min, followed by incubation with GELase (Epicentre, USA) at 43 °C for 45 min. The recovered DNA was drop dialyzed against Tris-EDTA for 4 h using a 0.1-μm

membrane. The dialyzed DNA was quantified and stored for nicking.

Prior to molecular nicking, the DNA was equilibrated at room temperature for 30 min and gently mixed with wide bore tips. In a 10-μl reaction system, 300 ng of equilibrated DNA was added to 7 units of Nt.BspQI (NEB, USA) nickase and 1 μl of nicking buffer and mixed. The nicking process was conducted in a thermal cycler at 37 °C for 2 h. The nicked DNA was incubated with 5 μl of labeling master mix containing 1.5 μl of labeling buffer (BioNano Genomics, USA), 1.5 μl of labeling mix (BioNano Genomics, USA), and 1 μl of Taq polymerase (NEB, USA) to flag specific motifs. The labeling process was conducted at 72 °C for 1 h. Each labeled DNA solution was mixed with 15 μl of Repair Master Mix containing 0.5 μl of 10 Thermo polymerase buffer (NEB, USA), 0.4 μl of 50× repair mix (BioNano Genomics, USA), 0.4 μl of 50 mM NAD+ (NEB, USA), 1.0 μl of Taq DNA polymerase (NEB, USA), and 2.7 μl of ultrapure water for nick repair. The repair reaction was conducted at 37 °C for 30 min, followed by the addition of 1 μl of stop solution (BioNano Genomics, USA) to stop the reaction. After ligating the nicks, the backbone of the labeled DNA was stained with IrysPrep DNA stain solution (BioNano Genomics, USA) at 4 °C overnight. The prepared samples were loaded onto Irys chips (BioNano Genomics, USA) and then applied to the chip nano-channels. The concentration time was 400 s to avoid over-staining or over-loading. The fluorescently labeled DNA was illuminated by the corresponding laser, and the signal was captured by an onboard electron microscope charge-coupled device camera (Fig. 1). The acquired images were converted to digital data with the AutoDetect software (BioNano Genomics, USA). In total, 517.874 Gb and 504.743 Gb of data were generated for *M. pentadactyla* and *M. javanica*, representing 178X and

177X coverage of their predicted genomes, respectively. Further methodological details available from protocols.io [9].

Genome assembly {2nd level heading}

All data were filtered by IrysView 2.4 using the following criteria: molecule lengths ≥150 kb and a label signal/noise ratio ≥ 3. The number of filtered molecules was approximately 1,360,730 for *M. pentadactyla* with a N50 length of 275.5 kb and 1,254,380 for *M. javanica* with a N50 length of 281.1 kb. The label density was 10.193/100 kb for *M. pentadactyla* and 10.151/100 kb for *M. javanica*. The distance between adjacent labels ranged from 0 kb to 833.609 kb for *M. pentadactyla* and 0 kb to 955.352 kb for *M. javanica*. During the detection process, two label sites that are near each other will be detected because they cannot be separated; therefore, the distance between these sites will be 0 bp. Simple tandem repeat areas with repeat units with only one restriction site were detected by the molecules. The statistical analysis revealed that the most common simple tandem repeat unit size was 4.3 kb, followed by 5.2 kb in *M. pentadactyla* and 4.6 kb and 3.4 kb in *M. javanica*. The total length of the repeat region accounted for 0.55% and 0.45% of the raw data. The RefAligner and Assembler packages in IrysView were used for *de novo* assembly. IrysView 2.4 can be obtained from BioNano Genomics [10]. The software requirements are as follows: Windows Python Runtime v2.7.5, Microsoft .Net 4.5.2, and Irys tools (RefAligner and Assembler [11]).

The assembly process comprised a molecule pairwise comparison, graph building and map refinement. In this work, the *P*-value thresholds were 1×10^{-9} for the pairwise assembly, 1×10^{-10} for the extension and refinement steps, and 1×10^{-11} for the final merging. The

false positive and false negative parameters were set to 1.5/100 kb and 0.15/100 kb. Finally, 2202 maps spanning 2.91 Gb of the genome were assembled for *M. pentadactyla* and 2096 maps spanning 2.85 Gb of the genome were assembled for *M. javanica*, with N50 lengths of 1.884 Mb and 1.972 Mb, respectively (Table 1). The largest *M. pentadactyla* fragment was approximately 14.21 Mb in size with 1354 label sites, and the largest *M. javanica* fragment was approximately 10.39 Mb in size with 1004 label sites (Fig. 2).

Genomics comparison {2nd level heading}

A whole-genome comparison between these two species was performed with RefAligner using a *P*-value of 1×10^{-9}. The results showed that 2196 maps covering 2.86 Gb from *M. pentadactyla* and 2088 maps covering 2.78 Gb from *M. javanica* could be mapped to one another with map rates of 97.544% and 98.282%, respectively. In total, 23,631 alignment blocks were generated. However, several reverse alignments were found in the blocks, suggesting that a series of large genome rearrangement events occurred during the divergence and evolution of these two species (Fig. 3).

Conclusion {2nd level heading}

Several phylogenetic investigations have been conducted in Manidae sequences using the *SRY* gene, *COX I* gene and whole mitochondrial sequence [12]. A series of genome projects for pangolins is ongoing. However, no genome-wide comparisons have been reported to date. Here, we present two optical maps of *M. pentadactyla* and *M. javanica* generated using the Irys system. These maps can serve as reliable references for further genome assembly. The comparison of the two maps revealed similarities and differences between *M. pentadactyla* and *M. javanica* and showed that potential genome rearrangement

events occurred during Manidae evolution. Our work implies that optical mapping provides reliable long-range linkage information for genome assembly and can be a suitable choice for convenient and low-coast genome-wide comparisons of highly related species. A new Pangolin genome has also recently been published [13] and this map can potentially aid in improving this reference.

Availability of supporting data and materials {1st level heading}

Datasets supporting this Data Note are deposited at the GigaScience repository, GigaDB [14]. Megabase DNA extraction and Irys NLRS DNA labeling and data collection protocols are also available via the protocols.io repository [9].

Competing interests {1st level heading}

Zhai Chaochao is an employee of Ultravision-tech.

Funding {1st level heading}

This work is supported by the National Nature Science Foundation of China (81403053 and 81503469), Guangdong Provincial Hospital of Chinese Medicine Special Fund (2015KT1817) and China Academy of Chinese Medical Sciences Special Fund for Health Service Development of Chinese Medicine.

Authors' contributions {1st level heading}

CSL, HZH and XJ designed the project. HZH, XSM, and GY prepared the samples. XJ, LBS,

and ZCC performed the experiments. LBS, XJ, XSM, and HZH analyzed the data. CSL, XJ, HZH, LBS, and QXH wrote the manuscript.

Acknowledgements {1st level heading}

We thank Chu Yang, Bai Rui and Ren Jian for useful suggestions on data interpretation.

References

1. Gaudin TJ, Emry RJ, Wible JR. The phylogeny of living and extinct Pangolins (Mammalia, Pholidota) and associated taxa: A morphology based analysis. J Mamm Evol. 2009;16(4):235–305. doi:10.1007/s10914-009-9119-9.
2. Teo ASM, Verzotto D, Yao F, Niranjan N, Hillmer AM. Single-molecule optical genome mapping of a human HapMap and a colorectal cancer cell line. GigaScience. 2015;4(1):1–6. doi:10.1186/s13742-015-0106-1.
3. Lam ET, Hastie A, Lin C, Ehrlich D, Das SK, Austin MD, et al. Genome mapping on nanochannel arrays for structural variation analysis and sequence assembly. Nat Biotech. 2012;30(8):771–6. doi:10.1038/nbt.2303.
4. Pendleton M, Sebra R, Pang AW, Ummat A, Franzen O, Rausch T, et al. Assembly and diploid architecture of an individual human genome via single-molecule technologies. Nature Methods. 2015;12(8):780–6. doi:10.1038/nmeth.3454.
5. Mostovoy Y, Levy-Sakin M, Lam J, Lam ET, Hastie AR, Marks P, et al. A hybrid approach for *de novo* human genome sequence assembly and phasing. Nature

Methods. 2016;13(7):587–90. doi:10.1038/nmeth.3865.

6. Vanburen R, Bryant D, Edger PP, Tang H, Burgess D, Challabathula D, et al. Single-molecule sequencing of the desiccation-tolerant grass *Oropetium thomaeum.* Nature. 2015;527(7579):508–11. doi:10.1038/nature15714.

7. Chen S, Xu J, Liu C, Zhu Y, Nelson DR, Zhou S, et al. Genome sequence of the model medicinal mushroom *Ganoderma lucidum.* Nat Commun. 2012;3(2):177–80. doi:10.1038/ncomms1923.

8. Grunwald A, Dahan M, Giesbertz A, Nilsson A, Nyberg LK, Weinhold E, et al. Bacteriophage strain typing by rapid single molecule analysis. Nucleic Acids Res. 2015;43(18) e117. doi:10.1093/nar/gkv563.

9. Huang Zhihai; Xu Jiang; Xiao Shuiming; Liao Baosheng; Gao Yuan; Zhai Chaochao; Qiu Xiaohui; Xu Wen; Chen Shilin. Comparative optical genome analysis of two Pangolin species Manis pentadactyla and Manis javanica. protocols.io. http://doi.org/10.17504/protocols.io.gaibsce; 2016

10. **Irysview 2.4:** http://bionanogenomics.com/products/irysview/

11. **Irys Tools:** http://bionanogenomics.com/support/software-updates/

12. Qin X, Dou S, Guan Q, Qin P, She Y. Complete mitochondrial genome of the *Manis pentadactyla* (Pholidota, Manidae): Comparison of *M. pentadactyla* and *M. tetradactyla.* Mitochondr DNA. 2012;23(1):37–8. doi:10.3109/19401736.2011.643881.

13. Choo SW, Rayko M, Tan TK, Hari R, Komissarov A, Wee WY, et al. Pangolin genomes and the evolution of mammalian scales and immunity. Genome Res. 2016.

doi:10.1101/gr.203521.115.

14. Huang Z, Xu J, Xiao S, Liao B, Gao Y, Zhai C, et al. Supporting data for "Comparative optical genome analysis of two Pangolin species: *Manis pentadactyla* and *Manis javanica*". GigaScience Database. 2016. http://doi.org/10.5524/100248.

{Figure legends}

Fig. 1 Irys raw images. Labeled high molecular weight DNA was linearized in the chip nano-channel. The restriction sites were digested with Nt.BspQI and labeled with fluorescence dNTP. The DNA backbone **(a,c)** and labels **(b,d)** were detected by EM CCD with blue (473 nm) and green (532 nm) lasers, respectively. Raw images in **a** and **b** are from *M. pentadactyla*; and in **c** and **d** from *M. javanica*. The raw molecule data were detected with Irys AutoDetect 2.1.4 from the raw images

Fig. 2 Assembled physical map. The physical maps were assembled and extended based on the similarity and overlap of molecules. The blue bars indicate the physical map and the green bars indicate the molecules. In the absence of amplification, the molecule coverage of each part of the physical map is very uniform. **a** and **b** are physical map examples of *M. pentadactyla* and *M. javanica*, respectively

Fig. 3 Physical map comparison of *M. pentadactyla* and *M. javanica.* The physical maps of *M. pentadactyla* and *M. javanica* were compared based on sequence similarity. The physical map of *M. pentadactyla* is shown at the top and *M. javanica* is shown at the bottom.

The line in the middle shows the links between similar regions. The comparison shows that the two species have high similarity at the physical map level (greater than 97% aligned to each other). However, some areas contained insertions/deletions or inversions (below)

Table legend

Table 1 Statistical analysis of the *M. pentadactyla* and *M. javanica* physical map data

		M. pentadactyla	***M. javanica***
**Raw data**			
	Quantity (Gb)	517.874 (178X)	504.743 (177X)
	Number of molecules	3,313,734	3,439,885
	Molecule N50 (kb)	216.3	212.4
	Label density (per 100 kb)	11.7	11.4
	Label SNR	11.7	10.8
**Filtered data**			
	Molecule length threshold (kb)	150	150
	Label SNR threshold	3	3
	Quantity (Gb)	360.483 (123X)	339.814 (119X)
	Number of molecules	1,360,730	1,254,380
	Molecule N50	275.5	281.1
	Label density	10.193	10.151
	Label SNR	12.7	11.4
**Assembly statistics**			

Total length (Gb)	2.91	2.85
Number of maps	2202	2094
Map N50 (Mb)	1.884	1.972
Average map length (Mb)	1.32	1.36
Maximum map length (Mb)	14.205	10.385
**Pairwise alignment**		
Number of aligned maps	2196	2088
Total aligned length (Mb)	4904.52	4716.849
Unique aligned length (Mb)	2861.22	2783.567

SNR signal-to-noise ratio

Figure 1 Click here to download Figure Fig. 1.png

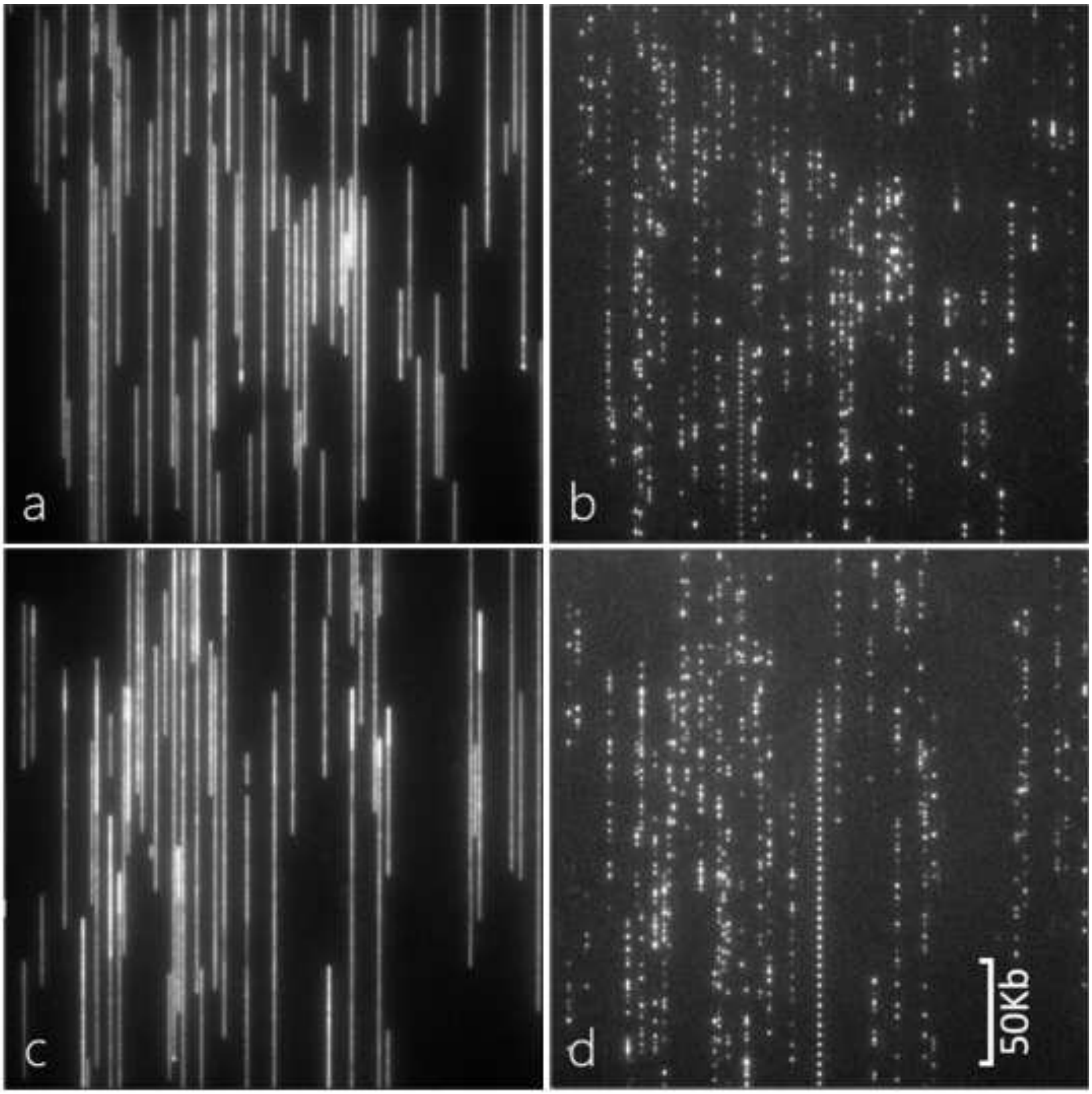

Figure 2

Click here to download Figure Fig. 2.png

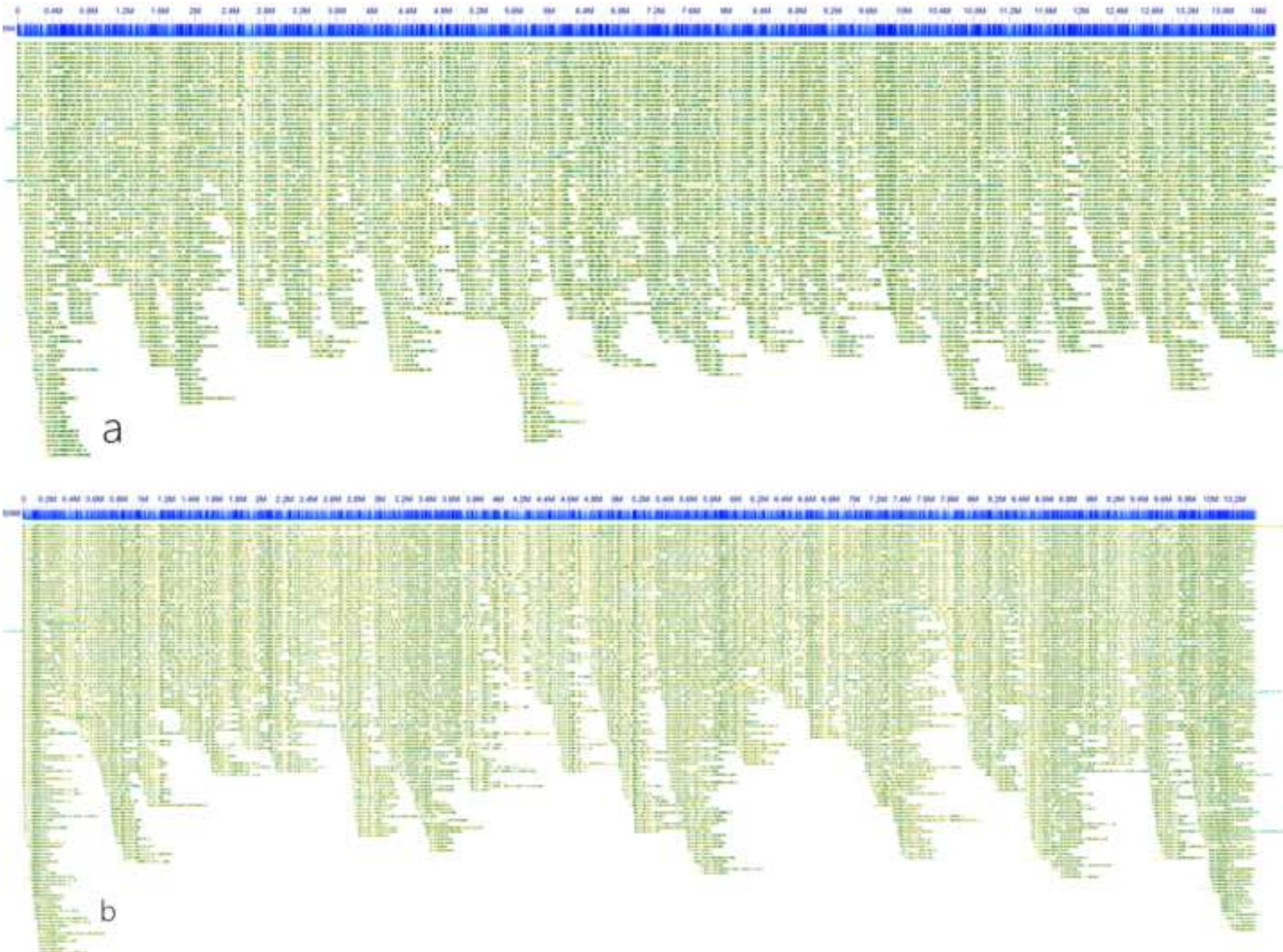

Figure 3 Click here to download Figure Fig. 3.png

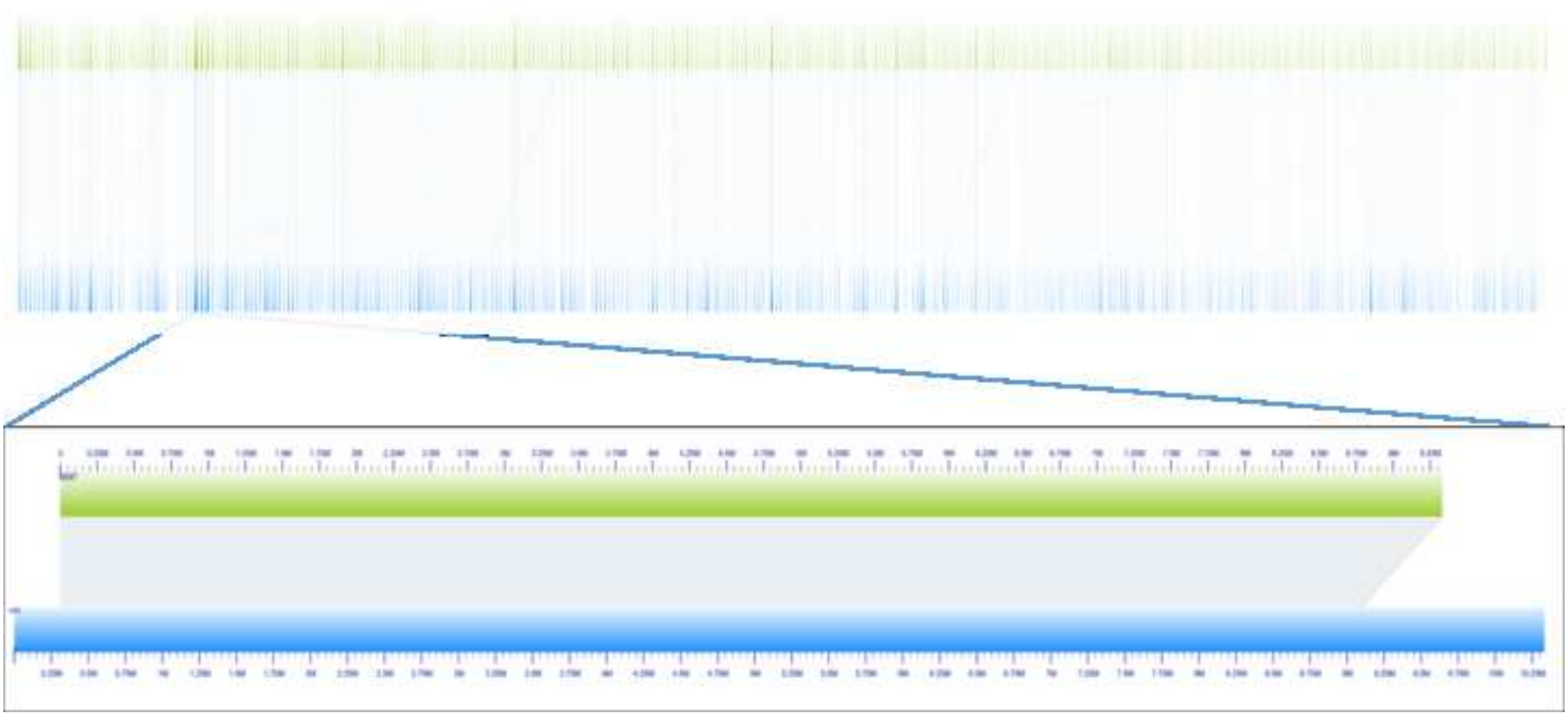

Personal Cover

Click here to download Personal Cover Zoo_Leipzig_-_Tou_Feng.jpg

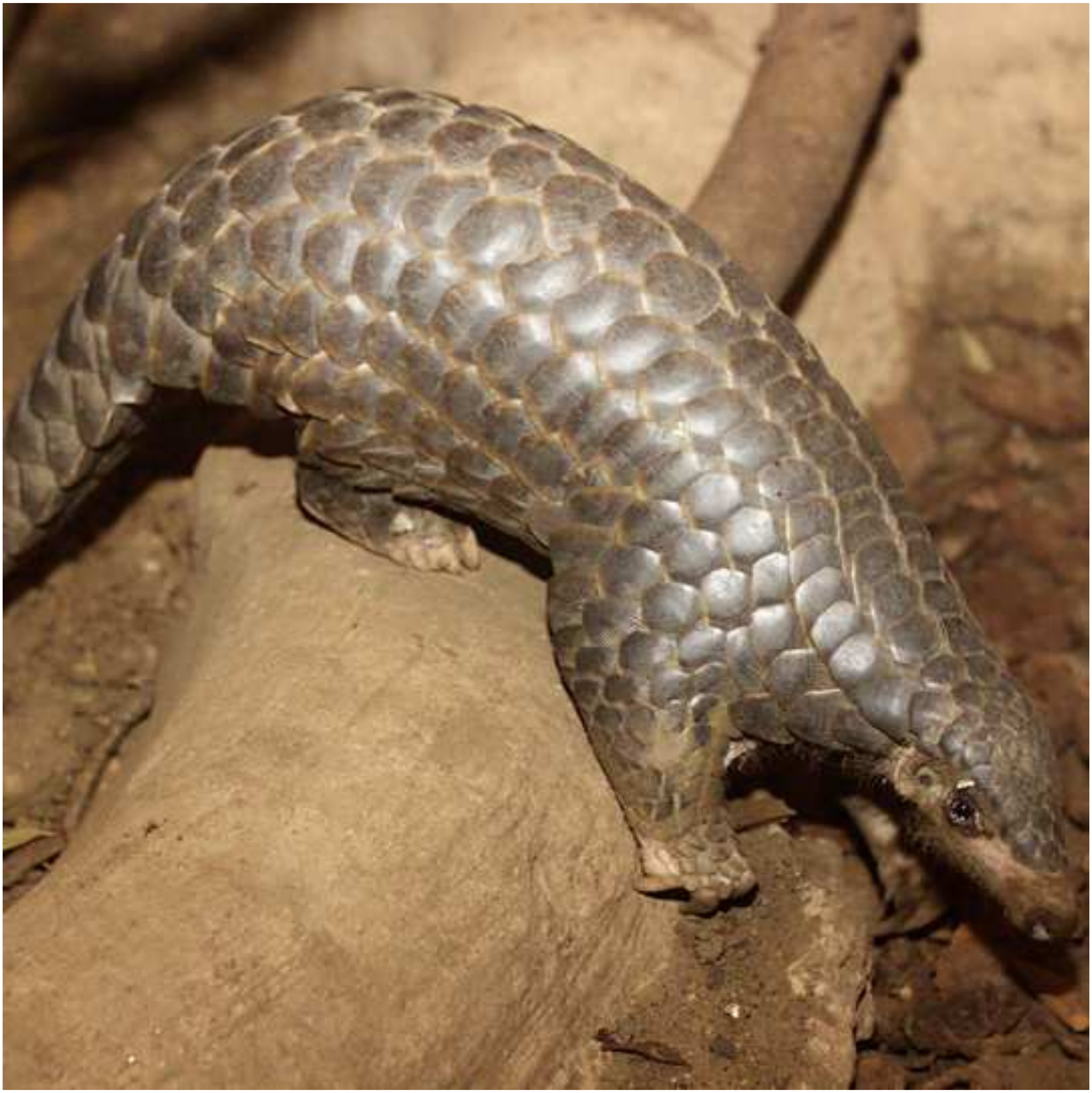

Supplement: Supplementary file 3 [file giw001_giga-d-16-00053_revision-2.pdf]
