# Supplementary material for: Comparative optical genome analysis of two pangolin species: Manis pentadactyla and Manis javanica
Source: Gigascience. 2016 Dec 29;5(1):1–5. doi: 10.1093/gigascience/giw001 (PMC6082621; doi:10.1093/gigascience/giw001)
Supplement: Supplementary file 4 [file giw001_response-to-reviewer-comments_original-submission.pdf]

Author's Response To Reviewer Comments

Close

Dear Dr. Nicole Nogoy,

We are grateful for the detailed comments from you and the reviewers. We have carefully considered all of the comments and revised our manuscript accordingly. Afterward, we have sent our manuscript to American Journal Experts for further language editing. Below please find point-by-point replies to the comments and detailed explanations of all changes (V1 indicates the originally submitted version and R1 indicates the revised version; all revisions were tracked in R1).

Reviewer #1: Huang et al. described generation and availability of genome mapping data for two endangered pangolin species. Genome mapping is a relatively new physical mapping platform useful for genome assembly/finishing and structural variation analysis. The datasets would likely be of interest to researchers studying pangolins and potentially those interested in applying genome mapping to large genomes. However, discussion of validation of the datasets was limited.

We thank reviewer #1 for the positive comments.

Comments:

1. Why was it of interest to compare these two particular pangolin species? Are there key phenotypic differences between them? What is known about their evolutionary relationship to each other?

Reply: Manis pentadactyla and Manis javanica both belong to order Pholidota and genus Manis and have been listed as endangered species in the IUCN Red list. Pangolins are the only mammals with large protective keratin scales covering their skin. Pangolins are nocturnal with poor vision and capture food using their slender and soft tongues. Although they belong to the same genus and live in similar areas, there are several morphological differences between these two species. First, the ratio of the length of the middle claws of the hind feet and fore feet in M. pentadactyla is less than 0.5, whereas the ratio in M. javanica is greater than 0.5. Second, the length of the protruding rim of the external ear in M. pentadactyla is greater than 10 mm, whereas the length in M. javanica is less than 10 mm. Third, the number of single flank scales of the edge tail in M. pentadactyla is less than 21, whereas the number in M. javanica exceeds 21. However, no significant differences in body weight and the length of the hind feet were detected between these two species (Wu, et al, Acta Theriologica Sinica, 2004). A phylogenetic analysis using the mitochondrial genomes revealed that M. pentadactyla and M. javanica were the most closely related pangolin species (Hassanin, et al, Comptes Rendus Biologies, 2015). In East Asia, M. pentadactyla and M. javanica are used in traditional medicines, but we do not think that these different species have the exact same uses. Thus, we wanted to compare these two species due to their special evolutionary statuses and potential medicinal uses.

2. What other sequence/genetic datasets are publically available for these two and related species?

Reply: At the time we submitted our manuscript, no published genome data were available. A genome research article about these two species was released online on the 30th of August (Choo, et al, Genome Research, 2016), but the scaffold N50 values in this study (approximatey200 kb) were not desirable for chromosome-wide comparisons.

3. If the information is available, please comment on the genetic heterogeneity of thesamples. Were these animals from the wild or in captivity?

Reply: Our samples are in captivity. All of our operations followed the Ethics Committee Orientation of Guangdong Provincial Hospital of Chinese Medicine. The pangolins were housed in Qingfeng Park Medicinal Animal Research Institute, Dongguan, Guangdong Province, which complies with the Domestication and Breeding License of the Wildlife under Special State Protection authorized by the Forestry Administration of Guangdong Province. From Choo's research, the genetic heterogeneity of M. javanica is 0.15% and the genetic heterogeneity of M. pentadactyla is 0.04% (Choo, et al, Genome Research, 2016).

4. The authors cited both studies that featured traditional optical mapping and ones that featured BioNano Genomics' genome mapping platform. It might be confusing for researchers unfamiliar with optical mapping technologies.

Reply: Thank you for noting this problem. We have specified the optical mapping platform that we used in the current study and updated the references as follows:
(1) Reference 3 in V1: "Tang H, Lyons E, Town CD. Optical mapping in plant comparative genomics. GigaScience. 2015;4(1):1-6. doi:10.1186/s13742-015-0044-y." was deleted.
(2) Reference 4 in R1: "Pendleton M, Sebra R, Pang AW, Ummat A, Franzen O, Rausch T, et al. Assembly and diploid architecture of an individual human genome via single-molecule technologies. Nature Methods. 2015;12(8):780-6.

doi:10.1038/nmeth.3454." replaced reference 5 in V1: "Chamala S, Chanderbali AS, Der JP, Lan T, Walts B, Albert VA, et al. Assembly and validation of the genome of the nonmodel basal angiosperm Amborella. Science. 2013;342(6165):1516-7. doi:10.1126/science.1241130."
(3) Reference 5 in R1: "Mostovoy Y, Levy-Sakin M, Lam J, Lam ET, Hastie AR, Marks P, et al. A hybrid approach for de novo human genome sequence assembly and phasing. Nature Methods. 2016;13(7):587-90. doi:10.1038/nmeth.3865." replaced reference 6 in V1: "Dong Y, Xie M, Jiang Y, Xiao N, Du X, Zhang W, et al. Sequencing and automated whole-genome optical mapping of the genome of a domestic goat (Capra hircus). Nat Biotech. 2013;31(2):135-41. doi:10.1038/nbt.2478."

5. The authors claimed that the maps would be reliable references for further analyses. However, there is generally little information or evidence on how reliable these datasets might be. It would be helpful to discuss whether the observed data was consistent with expectation.

5a. The authors mentioned that they collected 178X and 177X for the two species. What were the estimated genome sizes, and how were they determined? Were the assembly sizes close to the estimated genome sizes?

Reply: We calculated the data depth using the assembled physical map size. The size of the genome map generated from the BioNano Irys system was very close to the genome size estimated by sequence data or flow cytometry (the size of the genome map assembled from the BioNano Irys data was approximately 97% of the NGS assembly size (Vanburen, et al, Nature, 2015; Xiao, et al, BMC Genomics, 2015; Pendleton, et al, Nature Methods, 2015)). The genome map sizes in this study were 2.91 Gb (M. pentadactyla) and 2.85 Gb (M. javanica), which were comparable to Choo's research (Choo, et al, Genome Research, 2016).

5b. The authors mentioned that the distance between adjacent labels could be up to ~833 kb, even though the average label density was ~10 labels per 100 kb. Does this correspond to the centromere or known repetitive regions in the genome?

Reply: Thank you for your careful and thoughtful question. These areas may be centromeres or highly repetitive regions. Due to the uneven distribution of restriction sites, these areas may also be areas with no restriction sites. At present, we cannot confirm the locations of the specific regions because we do not have a high quality reference for comparison.

6. Part of Figure 3 seemed to be missing. Also, based on the figure, the two species (at least visually) seemed quite different, while the author claimed that the two species had high similarity. The author might consider updating the figure or explaining how one would interpret the alignment and the apparent discrepancy.

Reply: We apologize for the confusion caused by this figure. The alignment in Figure 3 from V1 has not been sorted. We sorted the alignment based on the alignment order and added one alignment example in this figure, which represented an approximately 10 Mb-length alignment between two similar regions of M. pentadactyla and M. javanica. The green bars in Figure 3 indicate the genome maps of M. pentadactyla and the blue bars indicate the genome maps of M. javanica.

7. The authors claimed that optical mapping was convenient and low-cost. Please briefly justify the statement.

Reply: A 200X sequencing depth is needed for any genome with an approximately 3 Gb nucleotide sequence, including shotgun libraries, mate-pair libraries and any other sequencing data from fosmids, BACs or linkage maps. The costs are approximately $20K for sequencing and $10K for library construction (only for shotgun and mate-pair, not including fosmids, BACs or linkage maps). Based on the species, the durations can range from 6 weeks to several months. In this study, only 3 chips were used for each species, which cost approximately $4.5K, and the whole process from DNA isolation to data analysis took only one week, which was obviously less than conventional NGS sequencing.

8. I would recommend using subheadings to separate the sections of the text under "Data description".

Reply: Thank you for the suggestion. We have added subheadings to separate the sections of the text under "Data description" to make the structure more explicit.
Paragraph 1-2: Background
Paragraph 3-4: DNA extraction, labelling and data collection
Paragraph 5: Genome assembly
Paragraph 6: Genomics comparison
Paragraph 7: Conclusion

9. I was not able to open the readme.txt file from the ftp site.

Reply: We apologize for the inconvenience. The readme.txt file contains brief descriptions of the uploaded files. The readme.txt file is attached here:

These data are associated with the manuscript "Comparative optical genome analysis of two Pangolin species: Manis pentadactyla and Manis javanica".

Authors:
Huang Zhihai1,*,&, Xu Jiang2,&, Xiao Shuiming2,&, Liao Baosheng2,3,&, Gao Yuan4, Zhai Chaochao3, Qiu Xiaohui1, Chen Shilin2,*
Abstract:
Background: The pangolin is a Pholidota mammal with large keratin scales protecting its skin. Two pangolin species (Manis pentadactyla and Manis javanica) have been recorded as critically endangered on the IUCN Red List of Threatened Species. Optical mapping constructs high-resolution restriction maps from a single-strand DNA molecule for genome analysis at the mega-base scale and to assist genome assembly. Here, we constructed restriction maps of M. pentadactyla and M. javanica using optical mapping to assist with genome assembly and analysis of these species.
Findings: Genomic DNA was nicked with Nt.BspQI, followed by labeling using fluorescent-labeled bases that were detected by the Irys optical mapping system. In total, 3,313,734 DNA molecules (517.847 Gb) for M. pentadactyla and 3,439,885 DNA molecules (504.743 Gb) for M. javanica were obtained, which corresponded to approximately 178X and 177X genome coverage, respectively. Qualified molecules (≥150 Kb with a label density of > 6 sites/100 kilobases) were analyzed using the de novo assembly program embedded in the IrysView pipeline. We obtained two maps that were 2.91 Gb and 2.85 Gb in size with N50s of 1.88 Mb and 1.97 Mb, respectively.
Conclusions: Optical mapping reveals large-scale structural information that is especially important for non-model genomes that lack a good reference. The approach has the potential to guide NGS-based de novo assembly. Our data provide a resource for Manidae genome analysis and references for de novo assembly. This note also provides new insights into Manidae evolutionary analysis at the genome structure level.

Data Description:
1. readme.txt: this introduction file
2. M.pentadactyla_RawMolecules.bnx: raw molecule data from M. pentadactyla generated from the BioNano Irys system
3. M.javanica_RawMolecules.bnx: raw molecule data from M. javanica generated from the BioNano Irys system
4. M.pentadactyla.cmap: physical map of M. pentadactyla assembled from raw molecules using the IrysSolve pipeline
5. M.javanica.cmap: physical map of M. javanica assembled from raw molecules using the IrysSolve pipeline

The data processing and data format instructions can be found at http://bionanogenomics.com/support/training/.

10. Under "Availability of supporting data and materials", it might be helpful to list what was deposited. Also, brief descriptions of the files would be helpful.

Reply: Thank you for your kind suggestion. We have included the data/file descriptions in the readme.txt.

11. There were grammatical issues; careful proofreading will be much needed.

Reply: Many thanks for the suggestion. We are sorry for our in-natural language. We have sent this version for language editing by native English speakers.

Reviewer #2: The Data Note by Zhihai et al. reports on construction of optical maps of two endangered species: Manis pentadactyla and Manis javanica. The authors isolated DNA from blood of the two species and analysed them using Bionano Irys instrument. Using standard software IrysView software they constructed optical maps of the species and performed their pairwise alignment.

The paper summarizes information on this dataset: statistics on data generated, assembled maps, alignment and common repeat sizes are reported. Figures represent screenshots from Irys software.

The reported dataset will be complimentary to NGS genomics data, that is being generated for these ogranisms. It can assist de novo genome assembly of Manis species or improving NSG-based Manis assembly with new optical mapping.

The manuscript needs extensive editing and improvement of English language, it is difficult to read. For example:
Abstract, line 27: "high-solution restriction maps" -> "high-REsolution restriction maps"
Abstract, line 31: "followed by labeling use certain fluorescent labeled bases" -> "followed by labeling usING certain fluorescentLY labeled bases"
Abstract, line 34: "which about 178X" -> "which CORRESPONDS TO about 178x"
Abstract, line 39: "especially for the non-model genome that without good reference" ->"especially important for non-model genome that lacks a good reference"
... and so on throughout the whole manuscript. Please improve.

Reply: Thank you for the comments and for noting the language problem. We have corrected our expression. We are sorry for our in-natural language. This version have been sent for language editing by native English speakers.

Close
