# Supplementary material for: Comparative optical genome analysis of two pangolin species: Manis pentadactyla and Manis javanica
Source: Gigascience. 2016 Dec 29;5(1):1–5. doi: 10.1093/gigascience/giw001 (PMC6082621; doi:10.1093/gigascience/giw001)
Supplement: Supplementary file 5 [file giw001_response-to-reviewer-comments_revision-1.pdf]

Author's Response To Reviewer Comments

Close

Dear Dr. Nicole Nogoy,

We are grateful for the detailed comments from you, the reviewers and the copy editors. We have carefully considered all of the comments and revised our manuscript accordingly. Below please find point-by-point replies to the comments and detailed explanations of all changes (R2 indicates the revised version; all revisions were tracked in R2).

1) Please carefully check the attached copy edited manuscript and address all comments by the copy editor.
Reply: We have carefully considered the all comments from editors, and responded them in R2.

2) Please cite the recently published Pangolin genome paper published in Genome Research, see: http://genome.cshlp.org/content/early/2016/09/13/gr.203521.115
Reply: Thank you for pointing it out and we have cited the paper in R2.

3) Also send us an image of the Pangolin species to use (public domain preferable).
Reply: The Pangolin images have been included in the "Pangolin images" word file.

Reviewer reports:
Reviewer #1: I believe all of my comments have been addressed, and the writing is much improved. I have no additional major comments.
One minor comment:
"Optical mapping constructs high-resolution restriction maps from a single-strand DNA molecule for genome analysis at the mega-base scale and to assist genome assembly" should be changed to "Optical mapping constructs high-resolution restriction maps from single DNA molecules for genome analysis at the mega-base scale and to assist genome assembly."
Reply: Thanks and we have changed the sentence as the reviewer's suggestion.

Close
