# Supplementary material for: Comparative optical genome analysis of two pangolin species: Manis pentadactyla and Manis javanica
Source: Gigascience. 2016 Dec 29;5(1):1–5. doi: 10.1093/gigascience/giw001 (PMC6082621; doi:10.1093/gigascience/giw001)
Supplement: Supplementary file 6 [file giw001_reviewer-1-report-(original-submission).pdf]

Reviewer Report

Title: Comparative optical genome analysis of two pangolin species: Manis pentadactyla and Manis javanica"

Version: Original Submission **Date:** 8/22/2016

Reviewer name: Ernest Lam

Reviewer Comments to Author:

Huang et al. described generation and availability of genome mapping data for two endangered pangolin species. Genome mapping is a relatively new physical mapping platform useful for genome assembly/finishing and structural variation analysis. The datasets would likely be of interest to researchers studying pangolins and potentially those interested in applying genome mapping to large genomes. However, discussion of validation of the datasets was limited.

Comments:
1. Why was it of interest to compare these two particular pangolin species? Are there key phenotypic differences between them? What is known about their evolutionary relationship to each other?
2. What other sequence/genetic datasets are publically available for these two and related species?
3. If the information is available, please comment on the genetic heterogeneity of the samples. Were these animals from the wild or in captivity?
4. The authors cited both studies that featured traditional optical mapping and ones that featured BioNano Genomics' genome mapping platform. It might be confusing for researchers unfamiliar with optical mapping technologies.
5. The authors claimed that the maps would be reliable references for further analyses. However, there is generally little information or evidence on how reliable these datasets might be. It would be helpful to discuss whether the observed data was consistent with expectation.
5a. The authors mentioned that they collected 178X and 177X for the two species. What were the estimated genome sizes, and how were they determined? Were the assembly sizes close to the estimated genome sizes?
5b. The authors mentioned that the distance between adjacent labels could be up to ~833 kb, even though the average label density was ~10 labels per 100 kb. Does this correspond to the centromere or known repetitive regions in the genome?
6. Part of Figure 3 seemed to be missing. Also, based on the figure, the two species (at least visually) seemed quite different, while the author claimed that the two species had high similarity. The author might consider updating the figure or explaining how one would interpret the alignment and the apparent discrepancy.
7. The authors claimed that optical mapping was convenient and low-cost. Please briefly justify the statement.
8. I would recommend using subheadings to separate the sections of the text under "Data description".
9. I was not able to open the readme.txt file from the ftp site.
10. Under "Availability of supporting data and materials", it might be helpful to list what was deposited. Also, brief descriptions of the files would be helpful.
11. There were grammatical issues; careful proofreading will be much needed.

Level of Interest

Please indicate how interesting you found the manuscript: An article of importance in its field

Quality of Written English

Please indicate the quality of language in the manuscript: Needs some language corrections before being published

Declaration of Competing Interests

Please complete a declaration of competing interests, considering the following questions:

- Have you in the past five years received reimbursements, fees, funding, or salary from an organisation that may in any way gain or lose financially from the publication of this manuscript, either now or in the future?
- Do you hold any stocks or shares in an organisation that may in any way gain or lose financially from the publication of this manuscript, either now or in the future?
- Do you hold or are you currently applying for any patents relating to the content of the manuscript?
- Have you received reimbursements, fees, funding, or salary from an organization that holds or has applied for patents relating to the content of the manuscript?
- Do you have any other financial competing interests?
- Do you have any non-financial competing interests in relation to this paper?

If you can answer no to all of the above, write 'I declare that I have no competing interests' below. If your reply is yes to any, please give details below.

I am an employee at BioNano Genomics, and I own company stock options. I am listed as an inventor of a patent owned by BioNano Genomics.

I agree to the open peer review policy of the journal. I understand that my name will be included on my report to the authors and, if the manuscript is accepted for publication, my named report including any attachments I upload will be posted on the website along with the authors' responses. I agree for my report to be made available under an Open Access Creative Commons CC-BY license (http://creativecommons.org/licenses/by/4.0/). I understand that any comments which I do not wish to be included in my named report can be included as confidential comments to the editors, which will not be published.

I agree to the open peer review policy of the journal
