# Supplementary material for: Comparative optical genome analysis of two pangolin species: Manis pentadactyla and Manis javanica
Source: Gigascience. 2016 Dec 29;5(1):1–5. doi: 10.1093/gigascience/giw001 (PMC6082621; doi:10.1093/gigascience/giw001)
Supplement: Supplementary file 8 [file giw001_reviewer-2-report-(original-submission).pdf]

Reviewer Report

Title: Comparative optical genome analysis of two pangolin species: Manis pentadactyla and Manis javanica"

Version: Original Submission **Date:** 8/30/2016

Reviewer name: Victor Guryev

Reviewer Comments to Author:

The Data Note by Zhihai et al. reports on construction of optical maps of two endangered species: Manis pentadactyla and Manis javanica. The authors isolated DNA from blood of the two species and analysed them using Bionano Irys instrument. Using standard software IrysView software they constructed optical maps of the species and performed their pairwise alignment.

The paper summarizes information on this dataset: statistics on data generated, assembled maps, alignment and common repeat sizes are reported. Figures represent screenshots from Irys software.

The reported dataset will be complimentary to NGS genomics data, that is being generated for these ogranisms. It can assist de novo genome assembly of Manis species or improving NSG-based Manis assembly with new optical mapping.

The manuscript needs extensive editing and improvement of English language, it is difficult to read. For example:

Abstract, line 27: "high-solution restriction maps" -> "high-REsolution restriction maps"
Abstract, line 31: "followed by labeling use certain fluorescent labeled bases" -> "followed by labeling usING certain fluorescentLY labeled bases"
Abstract, line 34: "which about 178X" -> "which CORRESPONDS TO about 178x"
Abstract, line 39: "especially for the non-model genome that without good reference" -> "especially important for non-model genome that lacks a good reference"

... and so on throughout the whole manuscript. Please improve.

Level of Interest

Please indicate how interesting you found the manuscript: An article whose findings are important to those with closely related research interests

Quality of Written English

Please indicate the quality of language in the manuscript: Not suitable for publication unless extensively edited

Declaration of Competing Interests

Please complete a declaration of competing interests, considering the following questions:

- Have you in the past five years received reimbursements, fees, funding, or salary from an organisation that may in any way gain or lose financially from the publication of this manuscript, either now or in the future?
- Do you hold any stocks or shares in an organisation that may in any way gain or lose financially from the publication of this manuscript, either now or in the future?
- Do you hold or are you currently applying for any patents relating to the content of the manuscript?
- Have you received reimbursements, fees, funding, or salary from an organization that holds or has applied for patents relating to the content of the manuscript?
- Do you have any other financial competing interests?
- Do you have any non-financial competing interests in relation to this paper?

If you can answer no to all of the above, write 'I declare that I have no competing interests' below. If your reply is yes to any, please give details below.

I declare that I have no competing interests

I agree to the open peer review policy of the journal. I understand that my name will be included on my report to the authors and, if the manuscript is accepted for publication, my named report including any attachments I upload will be posted on the website along with the authors' responses. I agree for my report to be made available under an Open Access Creative Commons CC-BY license (http://creativecommons.org/licenses/by/4.0/). I understand that any comments which I do not wish to be included in my named report can be included as confidential comments to the editors, which will not be published.

I agree to the open peer review policy of the journal
